# Viral Suppression, Viral Failure, and Safety Outcomes in Children and Adolescents With HIV on Dolutegravir in Europe and Thailand

**DOI:** 10.1093/cid/ciaf191

**Published:** 2025-04-11

**Authors:** Karen Scott, John O’Rourke, Charlotte Jackson, Luminita Ene, Luisa Galli, Tessa Goetghebuer, Cassidy Henegar, Christoph Königs, Magdalena Marczyńska, Lars Naver, Antoni Noguera-Julian, Paolo Paioni, Jose T Ramos, Birgitte Smith, Wipaporn Natalie Songtaweesin, Vana Spoulou, Nattakarn Tantawarak, Anna Turkova, Vani Vannappagari, Alla Volokha, Alastair Bamford, Hannah Castro, Elizabeth Chappell, Giorgia Dalla Valle, Caroline Foster, Sara Guillén Martín, Luis Manuel Prieto Tato, Thanyawee Puthanakit, Halyna Sherstiuk, Irina Shkurka, Sandra Soeria-Atmadja, Ali Judd, Siobhan Crichton, Intira Jeannie Collins, The European Pregnancy and Paediatric Infections Cohort Collaboration (EPPICC) study group, Elizabeth Chappell, Siobhan Crichton, Intira Jeannie Collins, Giorgia Dalla Valle, Charlotte Duff, Kate Edgar, Carlo Giaquinto, Charlotte Jackson, Ali Judd, Laura Mangiarini, John O’Rourke, Karen Scott, Claire Thorne, St Pierre Cohort, Tessa Goetghebuer, Marc Hainaut, Wivine Tremerie, Marc Delforge, Thomas Ulrik Hoffmann, Sannie Brit Nordly, Birgitte Smith, Christoph Königs, Vana Spoulou, Luisa Galli, Elena Chiappini, Catiuscia Lisi, Carlotta Montagnani, Elisabetta Venturini, Magdalena Marczyńska, Jolanta Popielska, Maria Pokorska-Śpiewak, Agnieszka Ołdakowska, Konrad Zawadka, Magdalena Pluta, Małgorzata Doroba, Luminita Ene, María José Mellado, Luis Escosa, Milagros García Hortelano, Talía Sainz, Carlos Grasa, Paula Rodríguez, Jose Tomás Ramos, Pablo Rojo, Luis Manuel Prieto Tato, Cristina Epalza, Alfredo Tagarro, Sara Domínguez, Álvaro Ballesteros, Marta Illán, Arantxa Berzosa, Sara Guillén, Beatriz Soto, María Luisa Navarro, Jesús Saavedra, Mar Santos, Elena Rincón, David Aguilera, Begoña Santiago, Beatriz Lázaro Martín, Andrea López Suárez, Amanda Bermejo, María Penín, Jorge Martínez, Katie Badillo, Ana Belén Jiménez, Adriana Navas, Eider Oñate, Itziar Pocheville, Elisa Garrote, Elena Colino, Olga Afonso, Jorge Gómez Sirvent, Mónica Garzón, Vicente Román, Raquel Angulo, Olaf Neth, Lola Falcón, Pedro Terol, Juan Luis Santos, Álvaro Vázquez, Begoña Carazo, Antonio Medina, Francisco Lendínez, Mercedes Ibáñez, Estrella Peromingo, María Isabel Sánchez, Beatriz Ruiz, Ana Grande, Francisco José Romero, Carlos Pérez, Alejandra Méndez, Laura Calle, Marta Pareja, Begoña Losada, Mercedes Herranz, Matilde Bustillo, Pilar Collado, José Antonio Couceiro, Leticia Vila, Consuelo Calviño, Ana Isabel Piqueras, Manuel Oltra, César Gavilán, Elena Montesinos, Marta Dapena, Beatriz Jiménez, Ana Gloria Andrés, Víctor Marugán, Carlos Ochoa, Ana Isabel Menasalvas, Eloísa Cervantes, Ignacio Bernardino, María Luisa Montes, Eulalia Valencia, Ana Delgado, Rafael Rubio, Federico Pulido, Otilia Bisbal, Alfonso Monereo Alonso, Juan Berenguer, Cristina Díez, Teresa Aldamiz, Francisco Tejerina, Juan Carlos Bernaldo de Quirós, Belén Padilla, Raquel Carrillo, Pedro Montilla, Elena Bermúdez, Maricela Valerio, Jose Sanz, Alejandra Gimeno, Miguel Cervero, Rafael Torres, Santiago Moreno, María Jesús Perez, Santos del Campo, Pablo Ryan, Jesus Troya, Jesus Sanz, Juan Losa, Rafael Gomez, Miguel Górgolas, Alberto Díaz, Sara de la Fuente, Jose Antonio Iribarren, Marıa Jose Aramburu, Lourdes Martinez, Ane Josune Goikoetxea, Sofia Ibarra, Mireia de la Peña, Víctor Asensi, Michele Hernandez, María Remedios Alemán, Ricardo Pelazas, María del Mar Alonso, Ana María López, Dácil García, Jehovana Rodriguez, Miguel Angel Cardenes, Manuel A Castaño, Francisco Orihuela, Inés Pérez, Mª Isabel Mayorga, Luis Fernando Lopez-Cortes, Cristina Roca, Silvia Llaves, Marıa Jose Rios, Jesus Rodriguez, Virginia Palomo, Juan Pasquau, Coral Garcia, Jose Hernandez, Clara Martinez, Antonio Rivero, Angela Camacho, Dolores Merino, Miguel Raffo, Laura Corpa, Elisa Martinez, Fernando Mateos, Jose Javier Blanch, Miguel Torralba, Piedad Arazo, Gloria Samperiz, Celia Miralles, Antonio Ocampo, Guille Pousada, Alvaro Mena, Marta Montero, Miguel Salavert, Maria Jose Galindo, Natalia Pretel, Joaquín Portilla, Irene Portilla, Felix Gutierrez, Mar Masia, Cati Robledano, Araceli Adsuar, Carmen Hinojosa, Begoña Monteagudo, Pablo Bachiller, Jesica Abadía, Carlos Galera, Helena Albendin, Marian Fernandez, Jose Ramon Blanco, Pere Soler-Palacín, Beatriz Álvarezand Santiago Pérez-Hoyos, Núria López, María Méndez, Clara Carreras, Borja Guarch, Teresa Vallmanya, Laura Minguell-Domingo, Olga Calavia, Lourdes García, Maite Coll, Berta Pujol, Valentí Pineda, Neus Rius, Núria Rovira, Joaquín Dueñas, Clàudia Fortuny, Anna Gamell, Antoni Noguera-Julian, Lars Navér, Sandra Soeria-Atmadja, Vendela Hagås, Johanna Rubin, Nora Einarsson, I A Abela, K Aebi-Popp, A Anagnostopoulos, M Battegay, M Baumann, E Bernasconi, D L Braun, H C Bucher, A Calmy, M Cavassini, A Ciuffi, P A Crisinel, K E A Darling, G Dollenmaier, A Duppenthaler, M Egger, L Elzi, J S Fehr, J Fellay, K Francini, H Furrer, C A Fux, H F Günthard, A Hachfeld, D H U Haerry, B Hasse, H H Hirsch, M Hoffmann, I Hösli, M Huber, D Jackson-Perry, C R Kahlert, O Keiser, T Klimkait, M Kohns, L Kottanattu, R D Kouyos, H Kovari, K Kusejko, N D Labhardt, Karoline Leuzinger, B Martinez de Tejada, C Marzolini, K J Metzner, N Müller, J Nemeth, D Nicca, J Notter, P Paioni, G Pantaleo, M Perreau, Ch Polli, E Ranieri, A Rauch, L P Salazar-Vizcaya, P Schmid, O Segeral, R F Speck, M Stöckle, P E Tarr, M Thanh Lecompte, A Trkola, N Wagner, G Wandeler, M Weisser, S Yerly, Rachaneekorn Nadsasarn, Chutima Saisaengjan, Patama Deeklum, Phattharapa Khamkhen, Lucksanapon Pitikawinwong, Nattakarn Tantawarak, Pope Kosalaraksa, Chanasda Kakkaew, Piangjit Tharnprisan, Hermione Lyall, Alasdair Bamford, Karina Butler, Katja Doerholt, Conor Doherty, Caroline Foster, Ian Harrison, Julia Kenny, Nigel Klein, Gillian Letting, Paddy McMaster, Fungai Murau, Edith Nsangi, Katia Prime, Andrew Riordan, Fiona Shackley, Delane Shingadia, Sharon Storey, Gareth Tudor-Williams, Anna Turkova, Intira Jeannie Collins, Claire Cook, Siobhan Crichton, Donna Dobson, Keith Fairbrother, M Diana Gibb, Ali Judd, Marthe Le Prevost, Nadine Van Looy, Helen Peters, Kate Francis, Claire Thorne, L Thrasyvoulou, S Welch, K Fidler, J Bernatoniene, F Manyika, G Sharpe, B Subramaniam, R Hague, V Price, J Flynn, N Klein, A Bamford, D Shingadia, K Grant, S Yeadon, S Segal, S Hawkins, M Dowie, S Bandi, E Percival, M Eisenhut, L Anguvaa, L Wren, T Flood, A Pickering, P McMaster, C Murphy, J Daniels, Y Lees, F Thompson, A Williams, B Williams, S Pope, S Libeschutz, L Cliffe, S Southall, A Freeman, H Freeman, S Christie, A Gordon, D Rosie Hague, L Clarke, L Jones, L Brown, M Greenberg, C Benson, A Riordan, L Ibberson, F Shackley, S Patel, J Hancock, K Doerholt, K Prime, M Sharland, S Storey, E G H Lyall, C Foster, P Seery, G Tudor-Williams, N Kirkhope, S Raghunanan, Julia Kenny, A Callaghan, A Bridgwood, P McMaster, J Evans, E Blake, A Yannoulias, T Kaleeva, Y Baryshnikova, S Soloha, N Bashkatova, I Raus, O Glutshenko, Z Ruban, N Prymak, G Kiseleva, Alla Volokha, Ruslan Malyuta, H Bailey

**Affiliations:** MRC Clinical Trials Unit at University College London, London, United Kingdom; MRC Clinical Trials Unit at University College London, London, United Kingdom; MRC Clinical Trials Unit at University College London, London, United Kingdom; HIV Department, “Dr. Victor Babes” Hospital for Infectious and Tropical Diseases, Bucharest, Romania; Infectious Disease Unit, Meyer Children's Hospital, IRCCS, Florence, Italy; Department of Health Sciences, University of Florence, Florence, Italy; Hospital St Pierre Cohort, Université libre de Bruxelles, Brussels, Belgium; Epidemiology and Real World Evidence, ViiV Healthcare, Durham, North Carolina, USA; Goethe University, University Hospital Frankfurt, Department of Pediatrics and Adolescent Medicine, Frankfurt, Germany; Department of Children's Infectious Diseases, Medical University of Warsaw, Warsaw, Poland; Department of Pediatrics, Karolinska University Hospital, Stockholm, Sweden; Department of Clinical Science, Intervention and Technology (CLINTEC), Karolinska Institutet, Stockholm, Sweden; Malalties Infeccioses i Resposta Inflamatòria Sistèmica en Pediatria, Servei de Malalties Infeccioses i Patologia Importada, Institut de Recerca Pediàtrica Sant Joan de Déu, Barcelona, Spain; Centro de Investigación Biomédica en Red de Epidemiología y Salud Pública (CIBERESP), Madrid, Spain; Departament de Cirurgia i Especialitats Medicoquirúrgiques, Facultat de Medicina i Ciències de la Salut, Universitat de Barcelona, Barcelona, Spain; Division of Infectious Diseases and Hospital Epidemiology, University Children's Hospital Zurich, Zurich, Switzerland; Hospital 12 de Octubre Instituto de Investigación Sanitaria (i+12), Madrid, Spain; CIBERINFEC, ISCIII, Madrid, Spain; Universidad Complutense, Madrid, Spain; Department of Pediatrics, Hvidovre University Hospital, Copenhagen, Denmark; Department of Pediatrics, and School of Global Health, Faculty of Medicine, Chulalongkorn University, Bangkok, Thailand; First Department of Pediatrics, “Agia Sofia” Children's Hospital, Athens, Greece; Department of Pediatrics, Faculty of Medicine, Khon Kaen University, Khon Kaen, Thailand; MRC Clinical Trials Unit at University College London, London, United Kingdom; Epidemiology and Real World Evidence, ViiV Healthcare, Durham, North Carolina, USA; Shupyk National Healthcare, University of Ukraine, Kyiv, Ukraine; MRC Clinical Trials Unit at University College London, London, United Kingdom; Great Ormond Street Hospital for Children NHS Foundation Trust, London, United Kingdom; UCL Great Ormond Street Institute of Child Health, London, United Kingdom; MRC Clinical Trials Unit at University College London, London, United Kingdom; MRC Clinical Trials Unit at University College London, London, United Kingdom; Fondazione Penta ETS, Padua, Italy; Department of Pediatric Infectious Diseases, Imperial College Healthcare NHS Trust, London, United Kingdom; Hospital Universitario de Getafe, Getafe, Spain; Hospital 12 de Octubre Instituto de Investigación Sanitaria (i+12), Madrid, Spain; CIBERINFEC, ISCIII, Madrid, Spain; Universidad Complutense, Madrid, Spain; Department of Pediatrics, and School of Global Health, Faculty of Medicine, Chulalongkorn University, Bangkok, Thailand; Department of Infectious Diseases, Dnipropetrovsk Regional Medical Center for Socially Significant Diseases, Dnipro, Ukraine; Center for the Prevention of HIV Infection/AIDS and Hepatitis, Chernihiv Regional Hospital, Chernihiv, Ukraine; Department of Clinical Science, Intervention and Technology (CLINTEC), Karolinska Institutet, Stockholm, Sweden; Malalties Infeccioses i Resposta Inflamatòria Sistèmica en Pediatria, Servei de Malalties Infeccioses i Patologia Importada, Institut de Recerca Pediàtrica Sant Joan de Déu, Barcelona, Spain; MRC Clinical Trials Unit at University College London, London, United Kingdom; Fondazione Penta ETS, Padua, Italy; Departament de Cirurgia i Especialitats Medicoquirúrgiques, Facultat de Medicina i Ciències de la Salut, Universitat de Barcelona, Barcelona, Spain; MRC Clinical Trials Unit at University College London, London, United Kingdom

**Keywords:** HIV, effectiveness, dolutegravir, children/adolescents, ART

## Abstract

**Background:**

Dolutegravir (DTG) is a preferred anchor antiretroviral therapy (ART) for children and adolescents with HIV (CAWH).

**Methods:**

We assessed the effectiveness and safety of DTG in CAWH aged 0–18 years at DTG start in routine care in Europe and Thailand, evaluating viral suppression (viral load [VL] <50 copies/mL), cumulative incidence and associated factors of viral failure (VF; confirmed VL ≥400 copies/mL) and safety outcomes.

**Results:**

Of 1230 CAWH on DTG, 49% were female. At DTG start, median (IQR) age was 14 (11–16) years, 10% were ART-naive, 49% ART-experienced/suppressed (VL <200 copies/mL), 13% ART-experienced/viremic (VL ≥200 copies/mL), and 28% ART-experienced/unknown VL. Median duration on DTG was 93 (49–163) weeks. Virall suppression was 88%–91% throughout follow-up. Cumulative incidence (95% CI) of VF at weeks 96 and 144 was 4.3% (3.1%–6.1%) and 8.3% (6.2%–11.1%). Increased risk of VF was associated with female sex, ART-experienced/viremic, advanced/severe immunosuppression, previous treatment failure, and region (*P* < .05, adjusting for age, sex and ART/VL status). The risk of VF was lower on DTG than CAWH on protease-inhibitor-based regimens (*P* < .001). Among 1146 with clinical data, 26 (2%) experienced 52 DTG-related adverse events, including 5 serious adverse events. Of 849 with laboratory data, 44 (5%) had 54 grade ≥3 events (<1 per 100 person-years). DTG discontinuation by weeks 96 and 144 was 5.0% (3.8%–6.7%) and 9.5% (7.5%–12.0%).

**Conclusions:**

DTG was well tolerated, with ∼90% virally suppressed <50 copies/mL. VF was low overall but was significantly higher in children/adolescents ART-experienced and viraemic at DTG start, requiring close monitoring.

## Teaser

Children and adolescents with HIV on dolutegravir (DTG) in Europe and Thailand had low risk of viral failure overall (8.3% by 144 weeks). However, among treatment-experienced children with viremia at DTG start, nearly one quarter experienced viral failure.

## Graphical Abstract

**Graphical Abstract:**
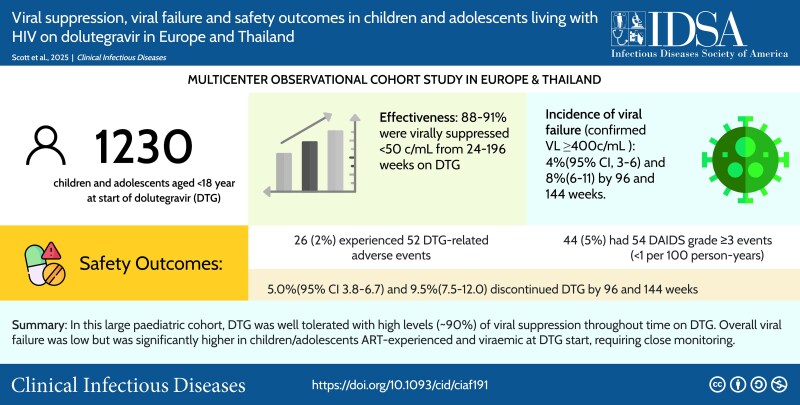
This graphical abstract is also available at Tidbit: https://tidbitapp.io/institutional-portal/clinical-infectious-diseases/tidbits/viral-suppression-viral-failure-and-safety-outcomes-in-children-and-adolescents-living-with-hiv-on-dolutegravir-in-europe-and-thailand-e2134500-ec6e-45cc-a103-6e224edab3d1/update

Dolutegravir (DTG), an integrase strand transfer inhibitor (INSTI), is a World Health Organization (WHO) preferred anchor drug for first- and subsequent-line antiretroviral therapy (ART) for children and adolescents with human immunodeficiency virus (HIV; CAWH) [1]. Dolutegravir has a high barrier to resistance and adult studies have reported long-term viral suppression (VS) [2, 3], low levels of viral failure (VF) (ranging from 0% to 8% at different durations of follow-up) [4], and good tolerability [5, 6]. While DTG has been rolled out to children globally, there remain limited comparable data on long-term outcomes in routine-care settings.

Two large pediatric clinical trials provided early data on DTG outcomes in CAWH. The ODYSSEY trial included 707 CAWH weighing 14 kg or more and aged less than 18 years, with 350 randomized to DTG as part of their first- or second-line ART. At 96 weeks on DTG, 81% were virally suppressed at fewer than 50 copies/mL (c/mL), treatment effects were similar among those receiving first- and second-line therapies [7]. Cumulative incidence of VF (confirmed viral load [VL] ≥400 c/mL) on DTG was 11.4% and 13.7% by 96 and 144 weeks, respectively, with a higher incidence on second-line ART [7]. In the CHAPAS-4 trial, 919 children aged 3–15 years experiencing first-line treatment failure were randomized to different second-line regimens. Among 229 children randomized to DTG, 83% were suppressed to less than 60 c/mL at 96 weeks [8]. Both trials reported low numbers of adverse events (AEs) and few DTG discontinuations.

Large pediatric observational cohorts in sub-Saharan Africa have reported 85%–93% VS (VL <400 or <1000 c/mL) on DTG in routine-care settings [9–11]. However, these studies had relatively short follow-up on DTG (<24 months), did not assess VF, and included limited safety data. European cohorts have reported good tolerability on DTG but were based on small samples (N = 150) [12, 13].

This study assesses the effectiveness and safety of DTG-based ART among CAWH in routine-care settings across Europe and Thailand, using data from the European Pregnancy and Pediatric Infections Cohort Collaboration (EPPICC).

## METHODS

Individual patient data from 15 observational cohorts across 14 countries were pooled using a modified HIV Cohorts Data Exchange Protocol (www.hicdep.org), as described elsewhere [14]. EPPICC (ClinicalTrials.gov ID: NCT04677842) has ethics approval from University College London (reference 17 493/001) and cohorts received local ethics approvals or waivers. Children and adolescents with HIV aged less than 18 years at DTG start were included. Time on DTG as part of a clinical trial was censored. The date of last follow-up varied by cohort (December 2020–May 2023).

### Outcomes on DTG

Effectiveness outcomes were as follows: (1) VS, defined as VL less than 50 c/mL at 24, 48, 96, 144 and 192 (±12) weeks after DTG start, in accordance with European guidelines [15]; (2) VF, defined as 2 consecutive VL results of  400 c/mL or greater or 1 VL result of 400 c/mL or greater followed by discontinuation of DTG within 4 months, after 24 weeks on DTG (the ≥400-c/mL threshold was used to align with definitions used in other pediatric HIV studies including the ODYSSEY trial). In sensitivity analysis, VF was defined as confirmed VL greater than 50 c/mL.

Safety outcomes were as follows: (1) clinical AEs causally related to DTG (as reported by the treating physician) and all serious AEs (SAEs); (2) laboratory abnormalities for lipids (total cholesterol, serum high-density lipoprotein [HDL], serum low-density lipoprotein [LDL], triglycerides), glucose (fasting plasma glucose [FPG] and non-FPG), other biochemistry (alanine aminotransferase, aspartate aminotransferase, alkaline phosphatase, gamma-glutamyl transferase, total bilirubin, plasma amylase, lipase, serum calcium, serum creatinine, serum phosphate), and hematology (absolute neutrophil count, hemoglobin, platelets); and (3) discontinuation of DTG, defined as stopping DTG for more than 30 consecutive days (all-cause and treatment-related [failure or toxicity]).

### Statistical Methods

#### Analysis of Effectiveness

Viral suppression analyses were restricted to CAWH in follow-up and on DTG for 24 weeks or more, with a VL measurement available at 1 or more time point (24–192 weeks). The percentage (95% binomial CI) with VS was estimated overall and by subgroups based on characteristics at DTG start: age (0 to <6, 6 to <12, 12 to <18 years), weight band (<20 kg, ≥20 kg), and ART/VL status (ART-naive, ART-experienced and viremic [VL ≥200 c/mL], ART-experienced and suppressed [defined as VL <200 c/mL to allow for transient viremia], and ART-experienced with unknown VL).

Viral failure analysis was restricted to CAWH on DTG with 2 or more VL measurements at 24 weeks or more after DTG start. Time to VF was estimated using Kaplan-Meier methods. Follow-up was censored at the earliest of last suppressed VL, death, or 7 days after DTG discontinuation. Cox proportional hazards models were used to explore associations between VF and characteristics at DTG start, including sex, age, ART/VL status, previous treatment failure, WHO immune stage for age [16] (none/mild, advanced/severe), prior AIDS diagnosis, duration on ART, and geographic region (United Kingdom/Ireland, Ukraine, Thailand, rest of Europe). The effect of nucleoside or nucleotide reverse transcriptase inhibitor (NRTI) backbone at DTG start was explored comparing tenofovir alafenamide (TAF), tenofovir disoproxil fumarate (TDF), and abacavir (ABC) within a subset of cohorts with availability of TAF. Children and adolescents receiving none of these NRTIs or 2 or more of these NRTIs simultaneously were excluded. Univariable and partially adjusted multivariable models (adjusted a priori for age, sex, and ART/VL status) were used as there were insufficient VF events for a fully adjusted analysis. Missing data were imputed for exposure variables with less than 30% missing using multiple imputation by chained equations using 20 datasets [17]. A sensitivity analysis used a complete case analysis without imputation.

#### Comparative Effectiveness of DTG

We compared time to VF on DTG with CAWH on protease inhibitor (PI)–based regimens in EPPICC. To maximize comparability of these groups, analysis was restricted to CAWH aged 6 to less than 18 years at start of PI or DTG combined with 2 or 3 NRTIs from 2012 onwards. Propensity score weighting was used to balance differences within each ART/VL subgroup in age, sex, ethnicity, and prior AIDS diagnosis, and in ART-experienced patients, time on ART, and previous treatment failure (a priori factors, insufficient numbers for additional factors). Propensity scores were estimated in those with 24 weeks or more on a regimen using logistic regression. Weighted Cox models (with robust standard errors to allow for clustering where CAWH contributed time on both PI- and DTG-based regimens) compared time to VF on DTG with PI-based regimens, overall and by ART/VL status at DTG/PI start. Follow-up was censored at last suppressed VL or discontinuation of DTG/PI for more than 30 days or 96 weeks. This was a complete case analysis with no imputation of missing data.

#### Analysis of Safety

Safety outcomes were assessed from DTG start until 30 days after discontinuation. Children and adolescents with HIV may have multiple episodes on DTG, while effectiveness analyses focused on the first episode all episodes were included in safety analyses.

Laboratory measurements were graded according to the Division of AIDS (DAIDS) 2014 criteria [18]. The DAIDS does not include HDL grading; therefore, US National Heart, Lung, and Blood Institute pediatric guidelines were used, with “acceptable” considered normal, “borderline” as grade 1, and “low” as grade 2 [19]. Laboratory events were defined according to the highest grade reached and included new events after DTG start, or an increase in severity for pre-existing abnormalities at DTG start. Frequency of events was described and rates of first events estimated per 100 person-years overall and over time from DTG start (<12, 12 to <24, ≥24 months). Person-years were censored at the start of the first event of that grade or higher or, if no event, then censored at the last visit. For laboratory markers with events in all time periods, Poisson models were used to assess differences in rates over time.

Cumulative incidence of all-cause DTG discontinuation was calculated using Kaplan-Meier methods. Treatment-related discontinuation was calculated with discontinuations for “other reasons” treated as a competing risk, using the Fine-Grey method [20]. Follow-up was censored at the last visit.

Analyses were conducted using Stata version 18 (StataCorp, College Station, TX, USA).

## RESULTS

Overall, 1230 CAWH ever on DTG were included: 606 (49%) females, 1019 (83%) with perinatally acquired HIV, 519 (42%) Black, and 382 (31%) from the United Kingdom/Ireland, 282 (23%) from Ukraine, 466 (38%) from the rest of Europe, and 100 (8%) from Thailand (Table 1).

**Table 1.** Demographic and Clinical Characteristics by ART and Viral Load Status at DTG Start

		ART and VL Status			
	Total (N = 1230)	Naive (n = 120)	ART Experienced, VL ≥200 Copies/mL (n = 163)	ART Experienced, VL <200 Copies/mL (n = 602)	ART Experienced, VL Unknown (n = 345)
Demographic characteristics					
Sex					
Male	597 (49%)	64 (53%)	73 (45%)	305 (51%)	155 (45%)
Female	606 (49%)	56 (47%)	90 (55%)	289 (48%)	171 (50%)
Unknown	27 (2%)	0 (<1%)	0 (<1%)	8 (1%)	19 (6%)
Ethnicity					
Black	519 (42%)	68 (57%)	72 (44%)	305 (51%)	74 (21%)
White	451 (37%)	15 (12%)	48 (29%)	181 (30%)	207 (60%)
Asian	130 (11%)	27 (22%)	18 (11%)	45 (7%)	40 (12%)
Other	105 (9%)	6 (5%)	23 (14%)	58 (10%)	18 (5%)
Unknown	25 (2%)	4 (3%)	2 (1%)	13 (2%)	6 (2%)
Region					
United Kingdom/Ireland	382 (31%)	33 (28%)	56 (34%)	232 (39%)	61 (18%)
Thailand	100 (8%)	26 (22%)	14 (9%)	23 (4%)	37 (11%)
Ukraine	282 (23%)	5 (4%)	26 (16%)	80 (13%)	171 (50%)
Rest of Europe^a^	466 (38%)	56 (47%)	67 (41%)	267 (44%)	76 (22%)
Characteristics at HIV diagnosis/ART initiation					
Age at HIV diagnosis (n = 1053), y	2 [0, 6]	11 [6, 16]	2 [1, 5]	2 [0, 5]	2 [0, 4]
Route of HIV acquisition					
Perinatal acquisition	1019 (83%)	63 (52%)	149 (91%)	522 (87%)	285 (83%)
Other	53 (4%)	33 (28%)	2 (1%)	12 (2%)	6 (2%)
Unknown	158 (13%)	24 (20%)	12 (7%)	68 (11%)	54 (16%)
Age at ART initiation (n = 1197), y	3 [1, 8]	15 [11, 16]	3 [1, 9]	2 [0, 7]	3 [1, 7]
Characteristics at start of DTG					
Age, y	14 [11, 16]	15 [11, 16]	15 [12, 17]	13 [11, 16]	14 [12, 16]
Age group					
0 to <6 y	69 (6%)	7 (6%)	16 (10%)	37 (6%)	9 (3%)
6 to <12 y	319 (26%)	34 (28%)	26 (16%)	173 (29%)	86 (25%)
12 to <18 y	842 (68%)	79 (66%)	121 (74%)	392 (65%)	250 (72%)
Duration on ART (n = 1077),^b^ y	9 [5, 12]	…	7 [4, 13]	9 [5, 12]	10 [6, 12]
Viral load (n = 851),^c^ copies/mL	40 [20, 532]	30 550 [1890, 85 756]	5663 [1285, 35 558]	38 [20, 40]	…
CD4% (n = 867)^d^	33 [26, 40]	20 [14, 30]	26 [18, 32]	36 [31, 42]	35 [29, 40]
CD4 count (n = 910),^d^ cells/mm^3^	710 [492, 972]	424 [242, 622]	521 [338, 791]	806 [619, 1036]	725 [528, 942]
BMI-for-age *z* score (n = 921)^d^	0.28 [−0.65, 1.16]	0.38 [−1.01, 1.22]	0.55 [−0.51, 1.21]	0.34 [−0.56, 1.25]	0.01 [−0.90, 0.75]
Weight band					
<20 kg	76 (6%)	8 (7%)	19 (12%)	38 (6%)	11 (3%)
≥20 kg	857 (70%)	87 (72%)	119 (73%)	479 (80%)	172 (50%)
Unknown	297 (24%)	25 (21%)	25 (15%)	85 (14%)	162 (47%)
WHO immune stage^d^					
None	663 (54%)	37 (31%)	77 (47%)	457 (76%)	92 (27%)
Mild	120 (10%)	18 (15%)	32 (20%)	51 (8%)	19 (6%)
Advanced	53 (4%)	17 (14%)	20 (12%)	9 (1%)	7 (2%)
Severe	74 (6%)	31 (26%)	29 (18%)	11 (2%)	3 (<1%)
Unknown	320 (26%)	17 (14%)	5 (3%)	74 (12%)	224 (65%)
Prior AIDS diagnosis					
No	951 (77%)	111 (92%)	125 (77%)	454 (75%)	261 (76%)
Yes	262 (21%)	9 (8%)	37 (23%)	141 (23%)	75 (22%)
Unknown	17 (1%)	0 (<1%)	1 (<1%)	7 (1%)	9 (3%)
Previous treatment failure	269 (22%)	3 (2%)	75 (46%)	128 (21%)	63 (18%)
Year of DTG initiation	2018 [2017, 2020]	2019 [2017, 2021]	2017 [2016, 2019]	2018 [2017, 2019]	2020 [2018, 2021]
Initial DTG regimen					
1 NRTI + DTG (3TC + DTG)	11 (<1%)	1 (<1%)	1 (<1%)	7 (1%)	2 (<1%)
≥2 NRTIs + DTG	1083 (88%)	117 (98%)	128 (79%)	519 (86%)	319 (92%)
PI + DTG	49 (4%)	1 (<1%)	7 (4%)	32 (5%)	9 (3%)
≥1 NRTI + PI + DTG	66 (5%)	1 (<1%)	18 (11%)	34 (6%)	13 (4%)
NNRTI + DTG	2 (<1%)	0 (<1%)	0 (<1%)	2 (<1%)	0 (<1%)
≥1 NRTI + NNRTI + DTG	9 (<1%)	0 (<1%)	3 (2%)	5 (<1%)	1 (<1%)
Other combination	10 (<1%)	0 (<1%)	6 (4%)	3 (<1%)	1 (<1%)
NRTI backbone (n = 1069)^e^					
ABC containing	707 (66%)	73 (62%)	71 (56%)	396 (78%)	167 (53%)
TDF containing	257 (24%)	32 (27%)	33 (26%)	63 (12%)	129 (41%)
TAF containing	80 (7%)	10 (9%)	20 (16%)	42 (8%)	8 (3%)
Other/mixed	25 (2%)	2 (2%)	3 (2%)	9 (2%)	11 (3%)
Duration of DTG exposure, wk	93 [49, 163]	106 [51, 171]	100 [47, 173]	108 [60, 182]	68 [30, 126]

Characteristics are summarized as n (%) or median [IQR]. Sample sizes are given for variables with incomplete data.

Abbreviations: ABC, abacavir; ART, antiretroviral therapy; BMI, body mass index; DTG, dolutegravir; HIV, human immunodeficiency virus; IQR, interquartile range; NNRTI, non-nucleoside reverse transcriptase inhibitor; NRTI, nucleoside reverse transcriptase inhibitor; PI, protease inhibitor; TAF, tenofovir alafenamide; TDF, tenofovir disoproxil fumarate; VL, viral load; WHO, World Health Organization; 3TC, lamivudine.

^a^Countries include Belgium, Denmark, Germany, Greece, Italy, Poland, Romania, Spain, Sweden, and Switzerland.

^b^ART-experienced only.

^c^Closest within 12 wk before and 1 wk after DTG start.

^d^Closest within ±12 wk of DTG start.

^e^Among those on 2 NRTIs + DTG.

At DTG start, the median (interquartile range [IQR]) age was 14 (11, 16) years; 120 (10%) were ART-naive, 163 (13%) ART-experienced and viremic, 602 (49%) ART-experienced and suppressed at less than 200 c/mL, and 345(28%) were ART-experienced with unknown VL. Among those who were ART-experienced, the median duration on ART at DTG start was 9 (5, 12) years.

Among those who were ART-naive at DTG start, the median (IQR) age was 15 (11, 16) years; a higher proportion had non-perinatal or unknown mode of transmission (48%), 26% had severe immunosuppression, but a lower proportion had a prior AIDS diagnosis (8%) compared with those who were ART-experienced (Table 1).

Among those taking 2 or more NRTIs plus DTG, 707 (66%) were on ABC, 257 (24%) on TDF, 80 (7%) on TAF, and 25 (2%) were receiving other/mixed combinations. Overall, the median (IQR) duration on DTG was 93 (49, 163) weeks.

### Effectiveness Outcomes

#### Viral Suppression

**Figure 1.**
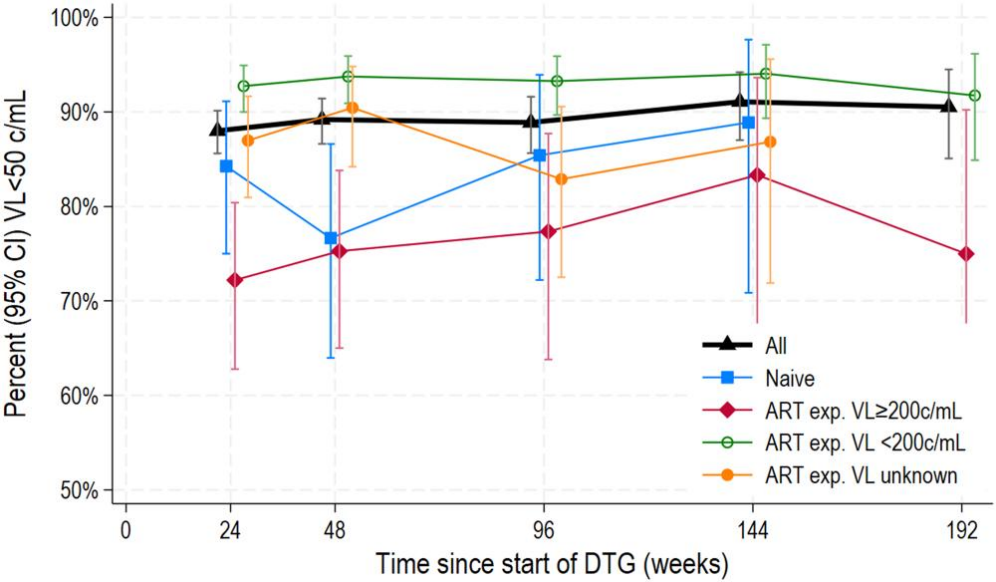
Viral suppression (<50 c/mL) over time, overall, and by ART experience/viral suppression at DTG start. Abbreviations: ART, antiretroviral therapy; c/mL, copies/mL; exp, experienced; DTG, dolutegravir; VL, viral load.

Overall, 88%–91% were suppressed at less than 50 c/mL at each time point on DTG (Figure 1, Table 2). The percentage suppressed was highest (92%–94%) among those who were ART-experienced/suppressed at DTG start and lowest (72%–83%) among those who were ART-experienced/viremic at DTG start. Viral suppression varied less by age and weight band at DTG start (Supplementary Tables 1 and 2, Supplementary Figure 1).

**Table 2.** Viral Suppression by Duration on DTG by ART and Viral Load Status at DTG Start

	ART and Viral Load Status at DTG Start														
	All			Naive			ART Experienced, VL ≥200 Copies/mL			ART Experienced, VL <200 Copies/mL			ART Experienced, VL Unknown		
	n/N^a^	%	(95% CI)	n/N^a^	%	(95% CI)	n/N^a^	%	(95% CI)	n/N^a^	%	(95% CI)	n/N^a^	%	(95% CI)
Viral load <50 copies/mL															
At 24 wk	734/834	88	(86, 90)	75/89	84	(75, 91)	78/108	72	(63, 80)	434/468	93	(90, 95)	147/169	87	(81, 92)
At 48 wk	611/685	89	(87, 91)	46/60	77	(64, 87)	67/89	75	(65, 84)	375/400	94	(91, 96)	123/136	90	(84, 95)
At 96 wk	408/459	89	(86, 92)	41/48	85	(72, 94)	41/53	77	(64, 88)	263/282	93	(90, 96)	63/76	83	(73, 91)
At 144 wk	245/269	91	(87, 94)	24/27	89	(71, 98)	30/36	83	(67, 94)	158/168	94	(89, 97)	33/38	87	(72, 96)
At 192 wk	153/169	91	(85, 94)	19	…	…	18/24	75	(53, 90)	100/109	92	(85, 96)	17	…	…

Abbreviations: ART, antiretroviral therapy; DTG, dolutegravir; VL, viral load.

^a^Patients in follow-up, still on DTG with VL data available at each time point (±12 wk) were included (if n ≥20).

#### Viral Failure

Among 777 CAWH meeting the criteria for VF analysis, 57 (7%) experienced VF at a median (IQR) of 79 (37, 123) weeks after DTG start. Of these, 3 of 57 (5%) never achieved VS of less than 400 c/mL after DTG start (all were ART-naive).

**Figure 2.**
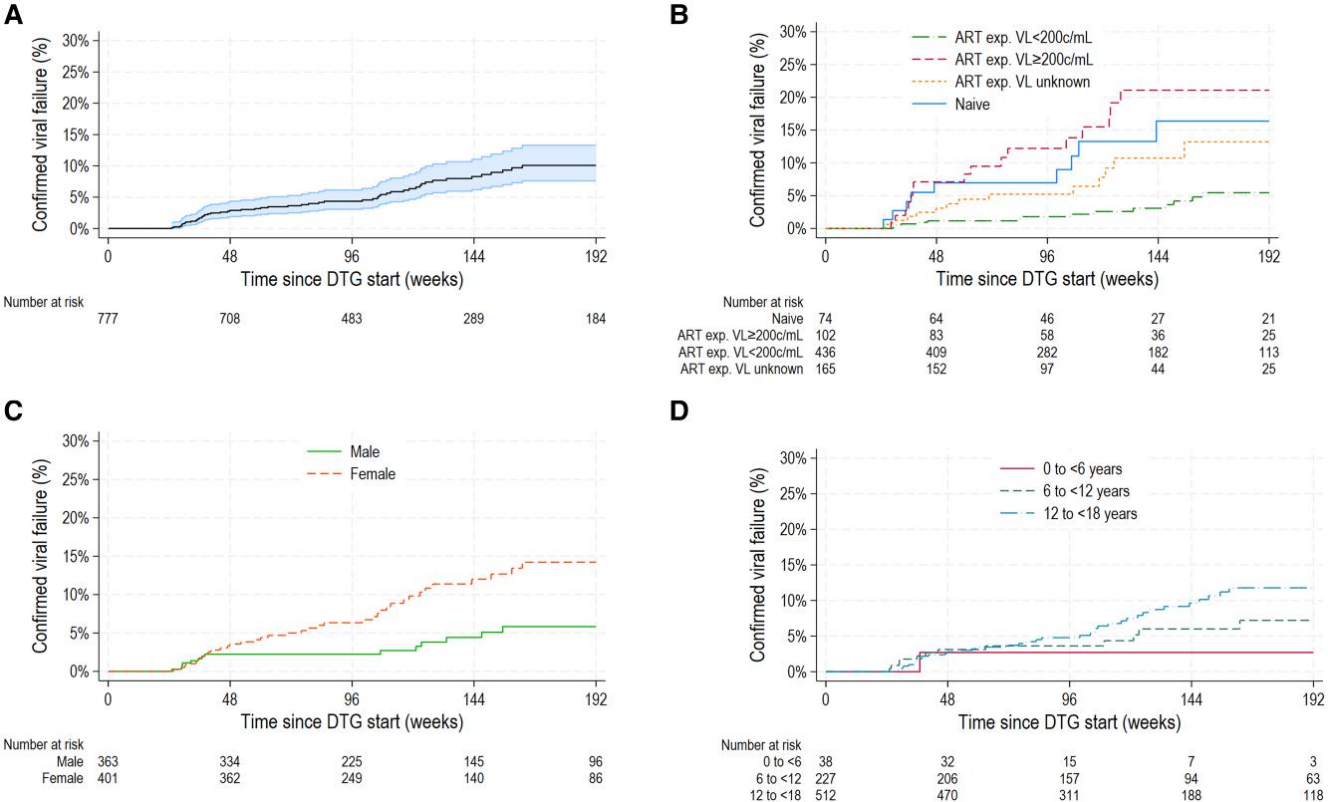
Time to viral failure on DTG (*A*) overall and by (*B*) ART and VL status, (*C*) sex, and (*D*) age group. Viral failure was defined as 2 consecutive VLs ≥400 c/mL after 24 weeks of treatment or 1 VL ≥400 c/mL after 24 weeks of treatment followed by discontinuation of DTG within 4 months. Incidence of failure was estimated using Kaplan-Meier methods. Follow-up was censored at the earliest of last suppressed VL or discontinuation of DTG in patients who did not experience viral failure. Analysis was restricted to patients with at least 24 weeks of follow-up after DTG start. Patients who initiated DTG in a trial are excluded. Abbreviations: ART, antiretroviral therapy; c/mL, copies/mL; DTG, dolutegravir; exp, experienced; VL, viral load.

The cumulative incidence (95% CI) of VF by 96 and 144 weeks was 4.3% (3.1%–6.1%) and 8.3% (6.2%–11.1%), respectively (Figure 2*A*). Incidence was lowest in those who were ART-experienced/suppressed at DTG start, at 1.8% (0.9%–3.8%) and 3.1% (1.6%–5.8%), and highest among those who were ART-experienced/viremic, at 12.1% (6.9%–21.1%) and 21.1% (13.2%–32.7%), respectively (Figure 2*B*). The corresponding estimates for those who were ART-naive were 7.0% (3.0%–15.9%) and 16.4% (8.5%–30.1%), respectively, and for those who were ART-experienced/unknown VL were 5.2% (2.6%–10.2%) and 10.7% (6.0%–18.9%), respectively.

In univariate analysis, VF was associated with sex, age, ART/VL group, immunosuppression status, previous treatment failure, and region (all *P* < .05) (Table 3). In models adjusted for sex, age, and ART/VL status at DTG start, female sex was associated with a 2-fold increase in hazard of failure (adjusted hazard ratio [aHR] = 2.30 [95% CI: 1.26–4.19] vs males; *P* = .006), as was previous treatment failure (aHR = 2.86 [1.57–5.18]; *P* < .001) (Table 3). There was a significant association with ART/VL status at DTG start: those who were ART-experienced/viremic had the highest hazard of VF (aHR = 4.38 [2.27–8.47]), followed by those who were ART-naive (aHR = 2.93 [1.32–6.51]) compared with those who were ART-experienced/suppressed (*P* < .001). There was also increased hazard with advanced/severe WHO immunosuppression (aHR = 2.22 [1.11–4.45] vs none/mild; *P* = .025) and region (*P* = .004). There was no association with age (*P* = .193) or duration on ART (*P* = .871). In analyses restricted to cohorts with access to TAF, there was no association with NRTI backbone (*P* = .227). Findings were similar using complete case analysis (Supplementary Table 4). The relationship between sex and VF was further explored, stratified by age at DTG start, and the increased hazard among females was only observed among adolescents (Supplementary Figure 2).

**Table 3.** Associations Between Participant Characteristics at DTG Start and Viral Failure

	Unadjusted			Adjusted for Age, Sex, and ART/VL Status at DTG Start		
	Hazard Ratio	(95% CI)	*P*	Hazard ratio	(95% CI)	*P*
Age (per year increase)	1.10	(1.00, 1.19)	.039	1.06	(.97, 1.15)	.193
Female sex (vs male)	2.66	(1.47, 4.79)	.001	2.30	(1.26, 4.19)	.006
Weight <20 kg (vs ≥20 kg)^a^	.92	(.23, 3.68)	.901	…	…	
Region						
United Kingdom/Ireland	1.00	…	.003	1.00	…	.004
Thailand	2.23	(.67, 7.45)		1.39	(.38, 5.12)	
Ukraine	.23	(.05, .98)		.18	(.04, .79)	…
Rest of Europe	.44	(.25, .79)		.41	(.22, .74)	…
ART and viral load status						
ART experienced, VL <200 copies/mL	1.00	…	<.001	1.00	…	<.001
ART experienced, VL ≥200 copies/mL	5.18	(2.73, 9.85)		4.38	(2.27, 8.47)	
Naive	3.18	(1.44, 7.02)		2.93	(1.32, 6.51)	
Advanced/severe immunosuppression (vs none/mild)	4.04	(2.28, 7.16)	<.001	2.22	(1.11, 4.45)	.025
Prior AIDS diagnosis (vs none)	.66	(.32, 1.34)	.245	.64	(.31, 1.31)	.221
Previous treatment failure (vs none)	4.12	(2.33, 7.29)	<.001	2.86	(1.57, 5.18)	<.001
Duration on ART (per year increase)^b^	1.03	(.97, 1.10)	.303	.99	(.93, 1.06)	.871
Backbone^c^						
ABC containing	1.00	…	.192	1.00	…	.227
TDF containing	1.32	(.31, 5.53)		.68	(.16, 2.99)	
TAF containing	2.27	(.93, 5.51)		2.08	(.84, 5.14)	

Multiple imputation was used to impute missing data for the following variables (n [%] missing): sex (n = 13 [2%]); weight band (n = 9 [1%]); viral suppression status at DTG start (n = 177 [23%]); WHO immune stage (n = 155 [20%]); prior AIDS diagnosis (n = 8 [1%]); duration on ART (n = 17 [2%]).

Abbreviations: ABC, abacavir; ART, antiretroviral therapy; CAWH, children and adolescents with HIV; DTG, dolutegravir; TAF, tenofovir alafenamide; TDF, tenofovir disoproxil fumarate; VL, viral load; WHO, World Health Organization.

^a^Omitted from adjusted model due to collinearity with age and ART/VL status.

^b^Among treatment-experienced CAWH at DTG start.

^c^Analysis of the backbone was in a subset of CAWH from cohorts where TAF was available.

In sensitivity analysis of VF using the more stringent threshold of confirmed VL of 50 c/mL or greater, the overall cumulative incidence was 10.0% (8.0%–12.5%) and 16.7% (13.7%–20.2%) by 96 and 144 weeks, respectively (Supplementary Table 3).

#### Comparative Effectiveness of DTG- vs PI-Based Regimen

A subset of 725 CAWH who started DTG at age 6 to <12 years on DTG + 2/3 NRTIs were compared with 572 who started a PI + 2/3 NRTIs regimen (274 [48%] on darunavir, 196 [34%] atazanavir, 100 [17%] lopinavir, 2 [<1%] fosamprenavir). Characteristics at DTG/PI start were similar, although those on DTG were more likely to start the regimen suppressed and without previous treatment failure (Supplementary Table 5). After propensity score weighting, the 2 groups were well balanced (Supplementary Table 6). The hazard of VF by 96 weeks was significantly lower on DTG- vs PI-based regimens (HR = .24; 95% CI: .16, .40; *P* < .001). When stratified by ART/VL status at DTG/PI start, the hazard of VF was significantly lower on DTG for ART-experienced subgroups (naive: HR = .39 [.12–1.24], *P* = .110; ART-experienced/suppressed: HR = .07 [.02–.33], *P* = .001; ART-experienced/viremic: HR = .31 [.16–.62], *P* = .001; ART-experienced/VL unknown: HR = .25 [.08–.81], *P* = .019) (Supplementary Figure 3). In sensitivity analysis restricted to those on ritonavir boosted darunavir (DRV/r)-based regimens compared with DTG, findings were similar, with a significantly lower hazard of VF on DTG (HR = .22; 95% CI: .13–.39; *P* < .001).

### Safety Outcomes on DTG

Of 1146 of 1230 (93%) CAWH on DTG with clinical data, 26 (2%) experienced 52 AEs related to DTG, including 5 SAEs. Four SAEs led to DTG discontinuation. An additional 5 SAEs occurred on DTG that were unrelated, or a causal relationship to DTG was unknown (Supplementary Table 7). There were no deaths.

Eleven CAWH experienced 15 neurological/neuropsychiatric AEs reported as possibly, probably, or definitively related to DTG: 3 SAEs (acute psychosis, headache and tiredness, headache during hospitalization for a central nervous system [CNS] lymphoma), headache (n = 5), insomnia (n = 2), dizziness/giddiness (n = 1), drowsiness (n = 1), depressed mood (n = 1), Bell's palsy (n = 1), and unspecified neurological event (n = 1). Five of these patients discontinued DTG during or 10 or fewer days after the event; 1 of the 15 events did not resolve (headache considered possibly related to DTG in a child with CNS lymphoma).

**Figure 3.**
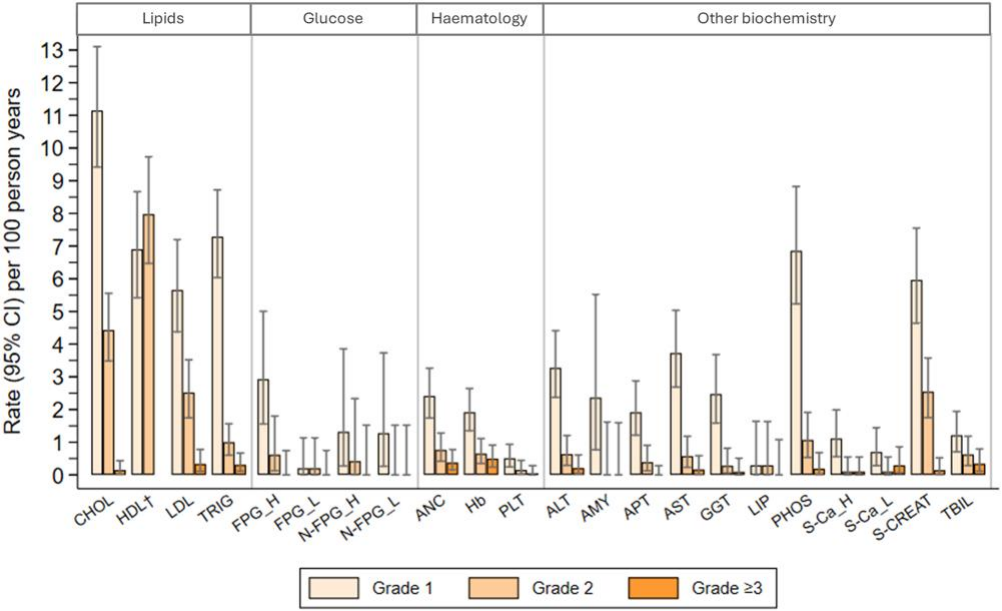
Rates of first grade 1, grade 2, and grade ≥3 laboratory events while on a DTG-based regimen stratified by laboratory marker. Event rates were calculated for episodes on DTG (CAWH could have >1 episode if they discontinued DTG for >30 days and then restarted DTG). Abbreviations: ALT, alanine aminotransferase; AMY, amylase; ANC, absolute neutrophil count; APT, alkaline phosphatase; AST, aspartate aminotransferase; CAWH, children and adolescents with HIV; CHOL, total cholesterol; DAIDS, Division of AIDS; DTG, dolutegravir; FPG_H, high fasting blood glucose; FPG_L, low fasting blood glucose; GGT, gamma-glutamyl transferase; Hb, hemoglobin; HDL, high-density lipoprotein; LDL, low-density lipoprotein cholesterol; LIP, lipase; N-FPG_H, high nonfasting blood glucose; N-FPG_L, low nonfasting blood glucose; PHOS, serum phosphate; PLT, platelets; S-Ca_H, high serum calcium; S-Ca_L, low serum calcium; S-CREAT, serum creatinine; TBIL, total bilirubin; TRIG, triglycerides.^†^ There are no DAIDS definitions for HDL abnormalities, instead the guidelines from the US Agency for Healthcare Research and Quality were used. “Borderline low” is presented as grade 1 and “low” as grade 2. There is no grade 3 or 4 category for HDL.

Among the 849 of 1230 (69%) CAWH with laboratory data, 44 (5%) experienced 54 DAIDS grade 3 or higher events. For all markers, the rates were less than 1 per 100 person-years (Figure 3, Supplementary Table 8). There were grade 3 or higher events in all time periods after DTG start (<12, 12 to <24, ≥24 months) for 4 markers (raised triglycerides, low absolute neutrophil count, low hemoglobin, raised bilirubin). There were significant differences in the rates of grade 3 or higher events over time for absolute neutrophil count and hemoglobin, with the highest rates in the first 12 months on DTG (Supplementary Figure 4). Rates of grade 2 laboratory AEs were highest for lipid markers and serum creatinine (Figure 3, Supplementary Table 8). Rates of grade 1 and 2 events were highest in the first 12 months for most markers (Supplementary Figure 4, Supplementary Table 9).

Overall, 95 (8%) CAWH discontinued DTG at a median (IQR) of 90 (36, 138) weeks, including 5 (5%) for VF, 17 (18%) for toxicity, 17 (18%) for treatment simplification/more effective treatment options, and 56 (59%) for other/unknown reasons (Supplementary Table 10). The cumulative incidence of discontinuation by 96 and 144 weeks was 5.0% (95% CI: 3.8%–6.7%) and 9.5% (7.5%–12.0%), respectively (Supplementary Figure 5). The incidence of treatment-related discontinuation (failure/toxicity) was 1.3% (0.7%–2.2%) and 1.7% (1.0%–2.8%), respectively.

## DISCUSSION

Our large study, spanning multiple countries in Europe and Thailand, included primarily treatment-experienced adolescents, with a median of 9 years on ART at DTG start. There were 3 key findings. First, DTG was generally well tolerated, with low rates of laboratory grade 3 or higher events and few clinical AEs causally related to DTG. The events reported included neurologically related symptoms, which were consistent with findings from previous studies [9, 12]. Second, high levels of effectiveness were observed overall, with approximately 90% VS throughout time on DTG. The overall incidence of VF was 8.3% by 144 weeks. However, among children/adolescents who were ART-experienced/viremic at DTG start, almost one quarter experienced VF. Third, in our comparative analysis of VF on DTG compared with PI-based regimens, there was significantly a lower hazard of VF on DTG by 96 weeks.

Large pediatric cohorts in sub-Saharan Africa have reported similarly high levels of VS, although we used a lower VL threshold (<50 c/mL vs <400 or <1000 c/mL in most African cohorts). In EPPICC, VS was highest among those who were ART-experienced and virally suppressed at DTG start, which constituted half of our cohort and may infer good adherence. In contrast, VS was lowest among those who were ART-experienced/viremic at DTG start, at 77% at less than 50 c/mL at 96 weeks, which is similar to the 73%–89% reported in comparable adult studies [21, 22] and is slightly lower than CAWH on DTG second-line ART in ODYSSEY (81% at <50 c/mL at 96 weeks in the ≥14-kg cohort) [7] and CHAPAS-4 [8] (83% at <60 c/mL at 96 weeks). This may be partly due to the inclusion of children/adolescents receiving DTG as part of their third- or subsequent-line treatment who were excluded from the above trials. While VS on DTG in EPPICC was high overall at approximately 90%, it is below the UNAIDS (Joint United Nations Programme on HIV and AIDS) target of 95% VS for all on ART by 2025 [23].

The low incidence of VF in our cohort was largely driven by very low incidence (∼3% at 144 weeks) among those who were ART-experienced and virally suppressed at DTG start; this population was not included in the ODYSSEY trial. Among those who were ART-naive at DTG start, 16.4% had VF by 144 weeks in EPPICC compared to 8.4% in ODYSSEY. Among those who were ART-experienced/viremic at DTG start, VF was 21.1% versus 17.9%, respectively. The higher incidence in our cohort may be partly due to the older age at DTG start (median: 14 years vs 12 years in ODYSSEY) and our ART-experienced/viremic group being more heavily treatment-experienced. Nonetheless, these findings highlight the need for close monitoring, particularly of outcomes following VF.

Few small observational studies [13, 24–26] have reported VF in CAWH on DTG, with estimates ranging from 4% to 17%, but these studies have used different definitions and time points for VF, making direct comparisons difficult. In our analysis, being female, being ART-experienced/viremic or ART-naive at DTG start, and having advanced or severe immunosuppression and previous treatment failure were associated with the highest hazard of VF. The 2-fold increase in hazard in females was unexpected, and on further analysis, this association appears to be driven by the adolescent group. While most studies to date have reported poorer virological outcomes in males [27, 28], a recent cohort study in Thailand reported poorer viral responses in females [29]. The reasons for this remain unclear and warrant further investigation.

Our third key finding was that our comparative analysis using propensity scoring methods showed a significantly lower risk of VF on DTG compared with PI-based regimens, consistent with findings from ODYSSEY, and supports the global roll-out of DTG. We also compared outcomes on DTG with DRV/r-based regimens only, and our findings were similar, with a significantly lower hazard of VF on DTG. This contrasts with findings from the CHAPAS-4 trial, where children on DRV/r- or DTG-based second-line ART had superior efficacy outcomes as compared with atazanavir or lopinavir-based regimens. However, the CHAPAS-4 primary outcome was VS at 96 weeks rather than confirmed VF used in our analysis. Also, our study did not have sufficient numbers to directly compare across individual PI-based regimens and we included children on second- and subsequent-line ART.

There are ongoing debates regarding excess weight gain after DTG start, with conflicting findings in adult studies and limited data in children/adolescents. Growth trends on DTG within this cohort were assessed in separate analyses, which showed weak evidence of greater increases in BMI-for-age *z* score (zBMI) in the 48 weeks after DTG start compared with the 48 weeks before. However, zBMI gains over 96 weeks on DTG were comparable to those observed in children/adolescents on PI-based regimens [30].

There are important study strengths and limitations to consider. Our cohort offers robust real-world evidence on the long-term safety and effectiveness of DTG in children/adolescents in routine-care settings and is one of the largest studies to date to estimate VF and associated factors. Due to the small number of VF events, we were limited to partially adjusted models, which may be subject to residual confounding. The effect of an NRTI backbone could not be fully assessed in this analysis due to the limited number of cohorts with access to TAF (see other analyses on NRTI backbone in EPPICC [31]). Outcomes after VF were not assessed due to insufficient follow-up time. Such data are needed to understand if children/adolescents are likely to re-suppress or remain viremic on DTG and the risk of accumulating INSTI resistance. Last, our cohort had limited data in young children (<6 years) on DTG. Data on longer-term outcomes across the age groups are needed to inform future care.

## Supplementary Material

ciaf191_Supplementary_Data

## Supplementary Data

Supplementary materials are available at *Clinical Infectious Diseases* online. Consisting of data provided by the authors to benefit the reader, the posted materials are not copyedited and are the sole responsibility of the authors, so questions or comments should be addressed to the corresponding author.

## Notes

***Author contributions***. All members of the Project Team participated in discussions about the study design, choice of statistical analyses, and interpretation of the findings, and were involved in the preparation and review of the final manuscript. Additionally, K. S. and J. O. drafted the manuscript, and K. S., J. O., and S. C. performed all statistical analyses. All members of the Writing Group were involved in the collection of data and interpretation of the findings.

***Acknowledgments***. The authors thank all the patients, families, and clinic staff who contribute to cohorts in the European Pregnancy and Pediatric Infections Cohort Collaboration (EPPICC). EPPICC is a collaborative study coordinated by the Penta Foundation (http://penta-id.org) and University College London.

***Financial support.*** This work was supported by ViiV Healthcare. The MRC Clinical Trials Unit at University College London (UCL) is supported by the Medical Research Council (UK) (program number: MC_UU_00004/03). Other EPPICC activities received industry funding from Gilead Sciences during the time this work was carried out.
