# Supplementary material for: Viral Suppression, Viral Failure, and Safety Outcomes in Children and Adolescents With HIV on Dolutegravir in Europe and Thailand
Source: Clin Infect Dis. 2025 Apr 11;81(4):e115–27. doi: 10.1093/cid/ciaf191 (PMC12596352; doi:10.1093/cid/ciaf191)
Supplement: ciaf191_Supplementary_Data [file ciaf191_supplementary_data.docx]

**Supplementary material**


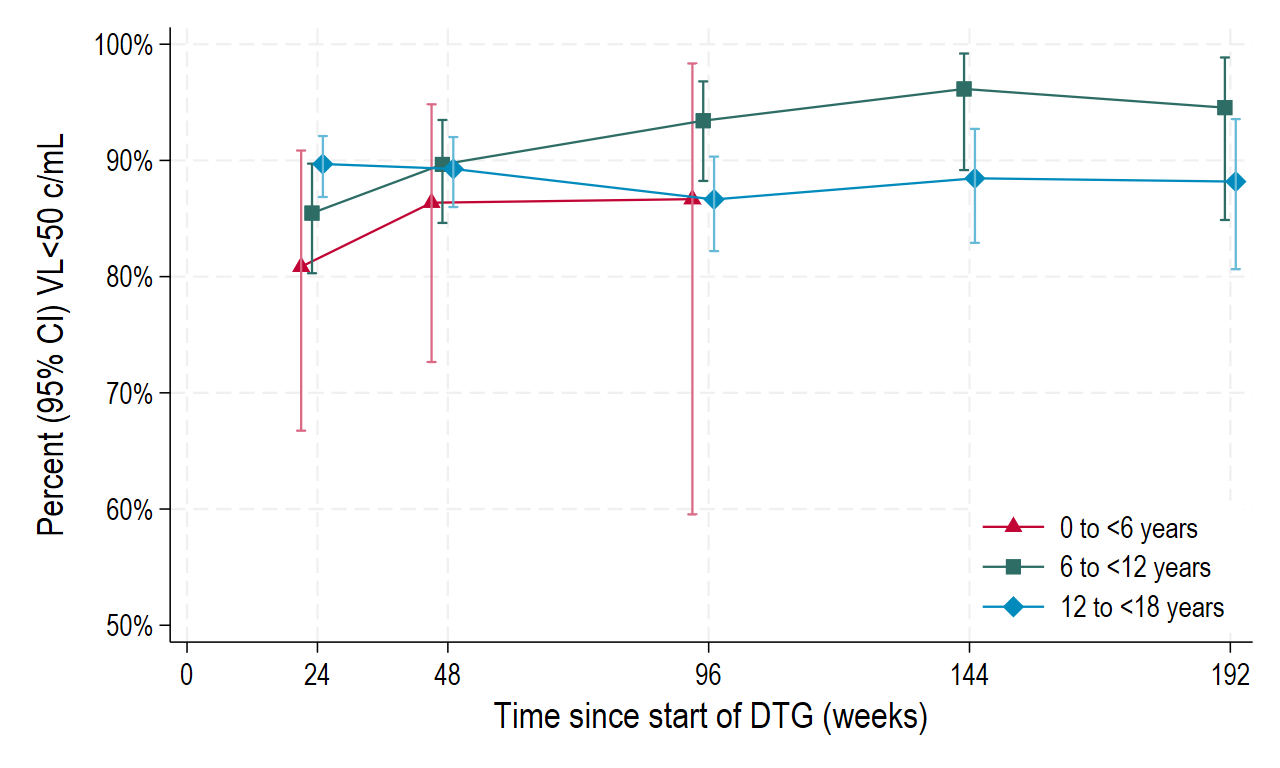

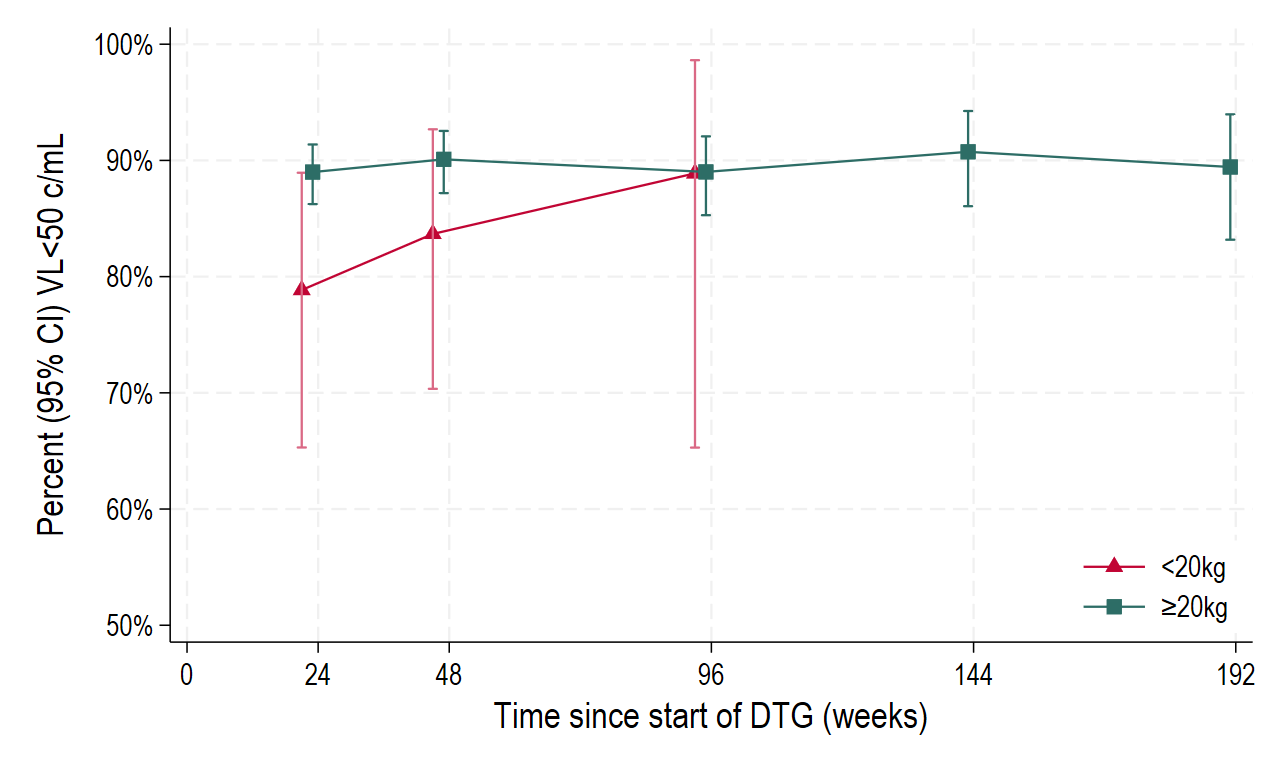


**Figure S1: Viral suppression (<50c/ml) over time by age group and weight-band at DTG start**

| **Table S1: Viral suppression by duration on DTG by age group at DTG start** | | | | | | | | | |
| --- | --- | --- | --- | --- | --- | --- | --- | --- | --- |
|  | **Age started DTG** | | | | | | | | |
|  | **0 to <6** | | | **6 to <12** | | | **12 to <18** | | |
|  | **n/N*** | **%** | **(95% CI)** | **n/N*** | **%** | **(95% CI)** | **n/N*** | **%** | **(95% CI)** |
| **Viral load <50 copies/mL** | | | | | | | | | |
| at 24 weeks | 38/47 | 81 | (67, 91) | 200/234 | 85 | (80, 90) | 496/553 | 90 | (87, 92) |
| at 48 weeks | 38/44 | 86 | (73, 95) | 182/203 | 90 | (85, 93) | 391/438 | 89 | (86, 92) |
| at 96 weeks | 15 |  |  | 142/152 | 93 | (88, 97) | 253/292 | 87 | (82, 90) |
| at 144 weeks | 9 |  |  | 75/78 | 96 | (89, 99) | 161/182 | 88 | (83, 93) |
| at 192 weeks | 4 |  |  | 52/55 | 95 | (85, 99) | 97/110 | 88 | (81, 94) |
| *Patients in follow up, still on a DTG with viral load data available at each time point (+/-12 weeks) were included (if n≥20). Abbreviations: ART, antiretroviral therapy; CI, confidence interval; mL, millilitre; DTG, dolutegravir | | | | | | | | | |

| **Table S2: Viral suppression by duration on DTG by weight band at DTG start** | | | | | | |
| --- | --- | --- | --- | --- | --- | --- |
|  | **Weight at DTG start** | | | | | |
|  | **<20kg** | | | **≥20kg** | | |
|  | **n/N*** | **%** | **(95% CI)** | **n/N*** | **%** | **(95% CI)** |
| **Viral load <50 copies/mL** | | | | | | |
| at 24 weeks | 41/52 | 79 | (65, 89) | 542/609 | 89 | (86, 91) |
| at 48 weeks | 41/49 | 84 | (70, 93) | 464/515 | 90 | (87, 93) |
| at 96 weeks | 18 |  |  | 316/355 | 89 | (85, 92) |
| at 144 weeks | 8 |  |  | 196/216 | 91 | (86, 94) |
| at 192 weeks | 4 |  |  | 127/142 | 89 | (83, 94) |
| *Patients in follow up, still on DTG with viral load data available at each time point (+/-12 weeks) were included (if n≥20). Abbreviations: ART, antiretroviral therapy; CI, confidence interval; kg, kilogram; mL, millilitre; DTG, dolutegravir | | | | | | |

| **Table S3: Cumulative incidence of viral failure≥50c/mL overall** | | | | |
| --- | --- | --- | --- | --- |
|  | **Number at risk** | **Total failures** | **Cumulative risk of failure (%)** | **95% CI** |
| **VF ≥50 c/mL** | | | | |
| DTG start | 777.0 | 0.0 | 0.0 |  |
| 24 weeks | 777.0 | 0.0 | 0.0 |  |
| 48 weeks | 685.0 | 49.0 | 6.4 | (4.9, 8.4) |
| 96 weeks | 458.0 | 72.0 | 10.0 | (8.0, 12.5) |
| 144 weeks | 270.0 | 99.0 | 16.7 | (13.7, 20.2) |
| 192 weeks | 170.0 | 105.0 | 18.9 | (15.6, 22.8) |


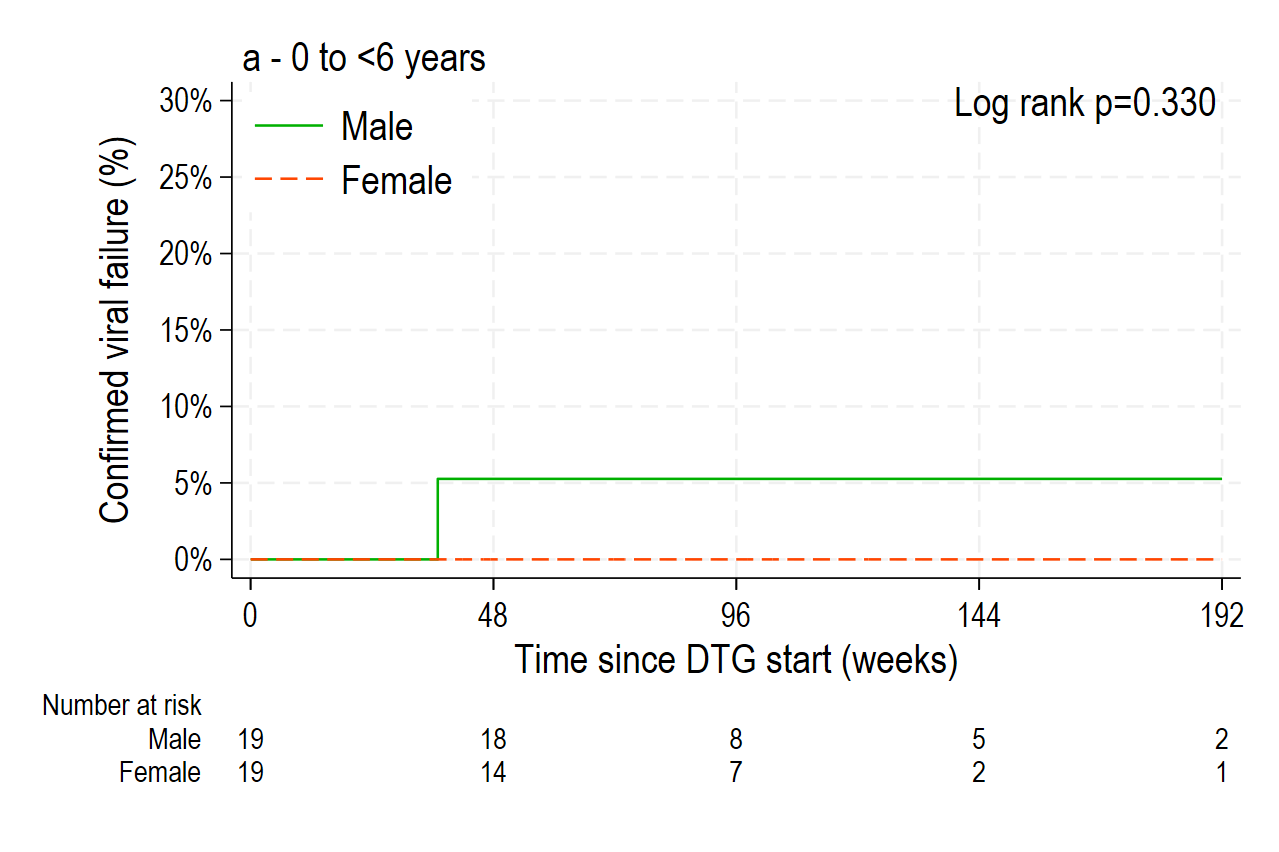

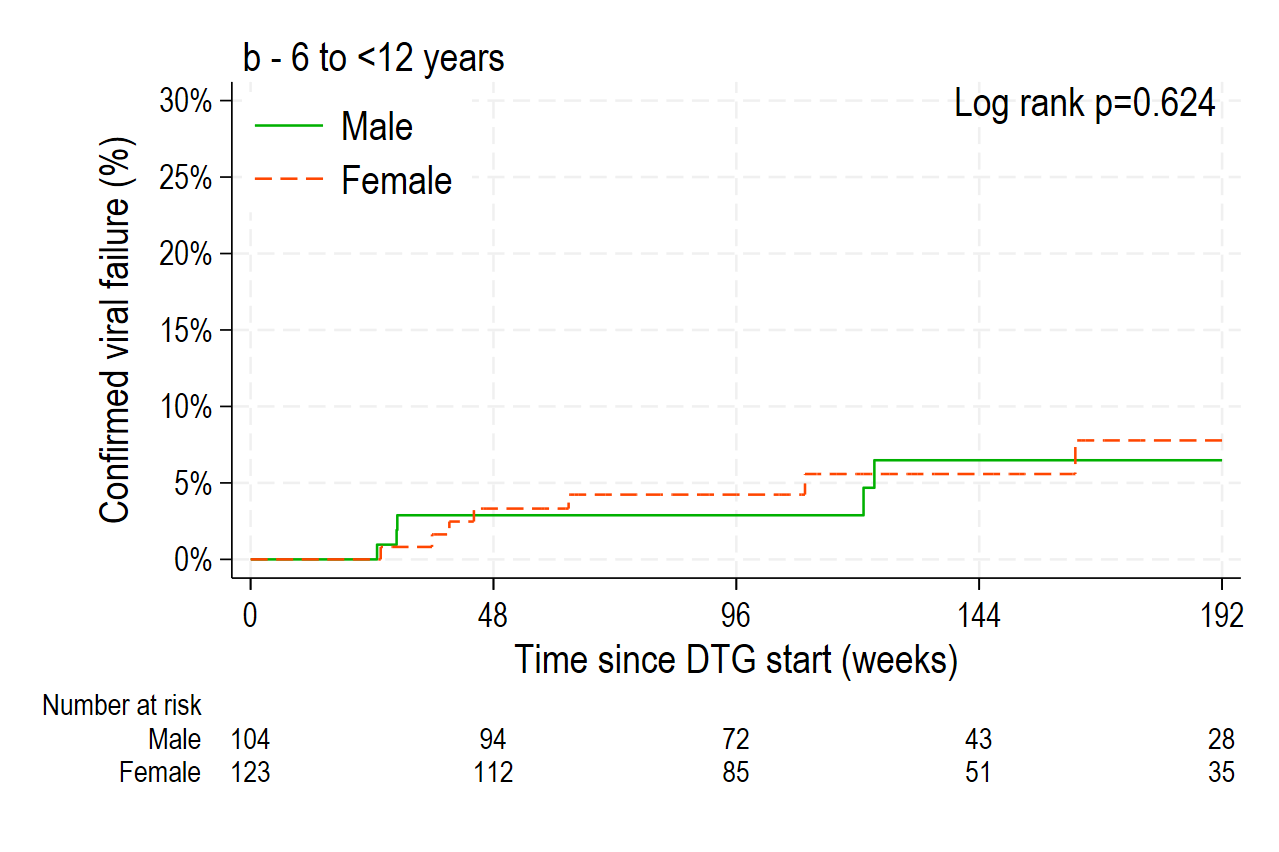

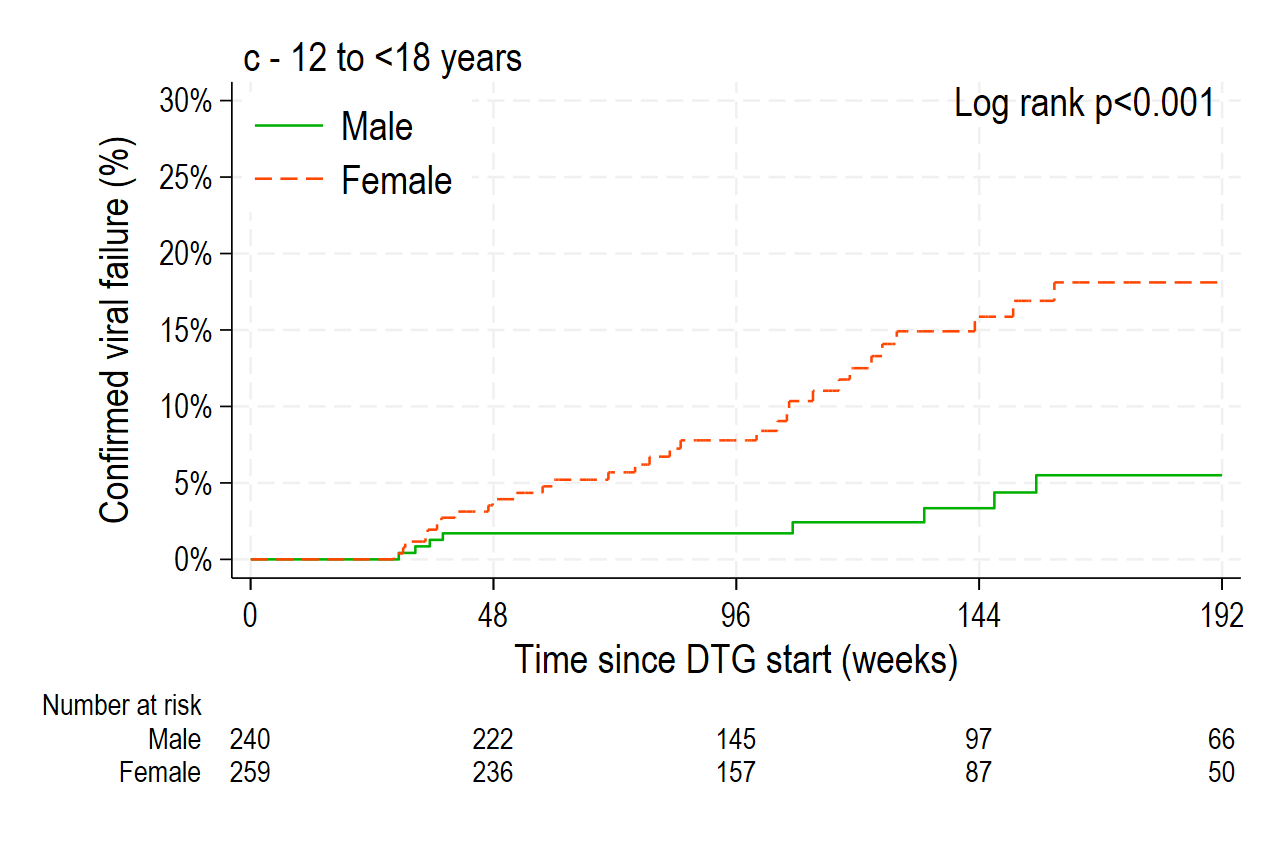


Viral failure was defined as 2 consecutive viral loads ≥400 copies/mL after 24 weeks of treatment OR 1 VL ≥400 copies/mL after 24 weeks of treatment followed by discontinuation of DTG within 4 months. Incidence of failure was estimated using Kaplan-Meier methods. Follow-up was censored at earliest of last suppressed viral load or discontinuation of DTG in patients who did not experience VF. Analysis was restricted to patients with at least 24 weeks follow up after DTG start. Patients who initiated DTG in a trial are excluded. Abbreviations: DTG, dolutegravir

**Figure S2: Time to viral failure on DTG by sex and age at DTG start (a) 0 to <6 years, (b) 6 to <12 years, and (c) 12 to <18 years**

| **Table S4: Associations between participant characteristics at DTG start and viral failure: sensitivity analysis using complete case set** | | | | | | |
| --- | --- | --- | --- | --- | --- | --- |
|  | Unadjusted | | | Adjusted for age, sex and ART/VL status at DTG start | | |
|  | Hazard ratio | 95% CI | p-value | Hazard ratio | 95% CI | p-value |
| Age (per year increase) | 1.10 | (1.00, 1.19) | 0.039 | 1.07 | (0.98, 1.17) | 0.123 |
|  |  |  |  |  |  |  |
| Female sex (vs male) | 2.66 | (1.47, 4.80) | 0.001 | 2.37 | (1.31, 4.28) | 0.004 |
|  |  |  |  |  |  |  |
| Weight band (vs ≥20kg)* |  |  |  |  |  |  |
| <20kg | 0.94 | (0.23, 3.92) | 0.937 |  |  |  |
|  |  |  |  |  |  |  |
| Region |  |  |  |  |  |  |
| UK/Ireland | 1.00 |  | 0.003 | 1.00 |  | 0.002 |
| Thailand | 2.23 | (0.67, 7.45) |  | 1.65 | (0.48, 5.64) |  |
| Ukraine | 0.23 | (0.05, 0.98) |  | 0.20 | (0.05, 0.86) |  |
| Rest of Europe | 0.44 | (0.25, 0.79) |  | 0.40 | (0.22, 0.73) |  |
|  |  |  |  |  |  |  |
| ART and viral load status |  |  |  |  |  |  |
| ART experienced, VL<200c/mL | 1.00 |  | <0.001 | 1.00 |  | <0.001 |
| ART experienced, VL≥200c/mL | 4.94 | (2.55, 9.59) |  | 3.95 | (2.00, 7.79) |  |
| Naive | 3.28 | (1.46, 7.36) |  | 2.94 | (1.30, 6.61) |  |
| ART experienced, VL unknown | 2.39 | (1.16, 4.93) |  | 2.33 | (1.13, 4.83) |  |
|  |  |  |  |  |  |  |
| Advanced/Severe immunosuppression (vs none/mild) | 4.17 | (2.27, 7.69) | <0.001 | 2.70 | (1.34, 5.45) | 0.006 |
|  |  |  |  |  |  |  |
| Prior AIDS diagnosis (vs none) | 0.65 | (0.32, 1.33) | 0.238 | 0.65 | (0.31, 1.34) | 0.240 |
|  |  |  |  |  |  |  |
| Previous treatment failure (vs none) | 3.02 | (1.62, 5.62) | <0.001 | 3.21 | (1.53, 6.74) | 0.002 |
|  |  |  |  |  |  |  |
| Duration on ART (per year increase)** | 0.97 | (0.90, 1.05) | 0.453 | 0.96 | (0.89, 1.04) | 0.313 |
|  |  |  |  |  |  |  |
| Backbone*** |  |  |  |  |  |  |
| ABC containing | 1.00 |  | 0.192 | 1.00 |  | 0.409 |
| TDF containing | 1.32 | (0.31, 5.53) |  | 0.70 | (0.16, 3.09) |  |
| TAF containing | 2.27 | (0.93, 5.51) |  | 2.06 | (0.84, 5.09) |  |
| Complete case analysis included CALHIV with complete data for each variable. Of the 777 eligible, the following n(%) were missing for: sex (n=13(2%)); weight band (n=9 (1%); Viral suppression status at DTG start (n=177(23%)); WHO immune-stage (n=155(20%)); prior AIDS diagnosis (n=8(1%)); duration on ART (n=17(2%)). *Due to correlation to weight band and age and ART/VL status adjusted analysis was not performed. **Among treatment experienced CALHIV at DTG start. ***Analysis of the backbone was in a subset of CALHIV from cohorts where TAF was available. Abbreviations: ABC, Abacavir; ART, antiretroviral therapy; CI, confidence interval; c/mL, copies per millilitre; DTG, dolutegravir; TAF, tenofovir alafenamide; TDF, tenofovir disoproxil fumarate; UK, United Kingdom, VL, viral load. | | | | | | |

| **Table S5: Characteristics of ART-experienced CALHIV at the start of DTG and PI based regimens included in VF analysis** | | |
| --- | --- | --- |
|  | **Anchor drug class** | |
|  | **DTG** | **PI** |
|  | **(n=725)** | **(n=572)** |
| **Demographic characteristics** | | |
| Sex |  |  |
| Male | 349 (48%) | 271 (47%) |
| Female | 376 (52%) | 301 (53%) |
| Ethnicity |  |  |
| Black | 362 (50%) | 384 (67%) |
| White | 239 (33%) | 127 (22%) |
| Other | 124 (17%) | 61 (11%) |
| Region |  |  |
| UK/Ireland | 241 (33%) | 393 (69%) |
| Thailand | 32 (4%) | 0 (0%) |
| Ukraine | 128 (18%) | 82 (14%) |
| Rest of Europe | 324 (45%) | 97 (17%) |
| Born abroad |  |  |
| No | 426 (59%) | 291 (51%) |
| Yes | 296 (41%) | 274 (48%) |
| Unknown | 3 (<1%) | 7 (1%) |
| Age at HIV diagnosis (years) | 2 [0, 6] | 3 [1, 7] |
| Age at ART initiation (years) | 4 [1, 9] | 6 [2, 11] |
| **Characteristics at start of regimen** | | |
| Age at regimen start (years) | 14 [11, 16] | 13 [10, 15] |
| Duration on ART (years) | 8 [4, 12] | 5 [0, 9] |
| Regimen start date (year) | 2018 [2017, 2019] | 2014 [2013, 2015] |
| Prior AIDS diagnosis |  |  |
| No | 571 (79%) | 456 (80%) |
| Yes | 154 (21%) | 116 (20%) |
| ART and viral load status at start of regimen |  |  |
| Naive | 78 (11%) | 139 (24%) |
| ART experienced, VL≥200c/mL | 82 (11%) | 121 (21%) |
| ART experienced, VL<200c/mL | 388 (54%) | 220 (38%) |
| ART experienced, VL unknown | 177 (24%) | 92 (16%) |
| Previous treatment failure |  |  |
| No | 512 (79%) | 255 (59%) |
| Previous treatment failure | 135 (21%) | 178 (41%) |
| NRTI backbone |  |  |
| No TDF/TAF | 541 (75%) | 367 (64%) |
| TDF | 124 (17%) | 190 (33%) |
| TAF | 60 (8%) | 15 (3%) |
| CD4 count (cells/mm³) | 735 [527, 960] | 660 [395, 940] |
| Characteristics are summarised as n (%) or median [IQR]. Abbreviations: 3TC, lamivudine; AIDS, acquired immunodeficiency syndrome; ART, antiretroviral therapy; DTG, dolutegravir; IQR, interquartile range; NRTI, Nucleoside Reverse Transcriptase Inhibitor; PI, protease inhibitor; TAF, tenofovir alafenamide; TDF, tenofovir disoproxil fumarate; UK, United Kingdom; VL, viral load. 52 CALHIV on eligible regimens were excluded from analysis due to unknown sex (n=11), prior AIDS event (n=11) or unknown ethnicity (n=30) | | |

| **Table S6: Covariate balance before and after weighting patients on DTG and PI regimens and who were included in comparative analyses of viral failure** | | | | | |
| --- | --- | --- | --- | --- | --- |
|  | **DTG based regimen** | **PI based regimen** |  |  |  |
|  | **Mean or % (± SD)** | **Mean or % (± SD)** | **Weighted mean** | **Unweighted standardised difference*** | **Weighted standardised difference*** |
| **All** |  |  |  |  |  |
| Male sex | 0.48±0.50 | 0.47±0.50 | 0.44 | 0.02 | 0.09 |
| Black ethnicity | 0.50±0.50 | 0.67±0.47 | 0.53 | -0.34 | -0.06 |
| Age at regimen start (years) | 13.14±3.01 | 12.22±3.15 | 12.99 | 0.30 | 0.05 |
| Prior AIDS diagnosis | 0.21±0.41 | 0.20±0.40 | 0.21 | 0.02 | 0.00 |
| Previous treatment failure | 0.21±0.41 | 0.41±0.49 | 0.23 | -0.50 | -0.05 |
| Time since ART start | 7.85±4.84 | 5.71±4.92 | 7.70 | 0.44 | 0.03 |
| ART naive | 0.11±0.31 | 0.24±0.43 | 0.11 | -0.44 | -0.01 |
| ART exp., VL>=200c/mL | 0.11±0.32 | 0.21±0.41 | 0.13 | -0.31 | -0.04 |
| ART exp., VL<200c/mL | 0.54±0.50 | 0.38±0.49 | 0.54 | 0.30 | -0.02 |
| UK/Ireland* | 0.33±0.47 | 0.69±0.46 | 0.64 | -0.75 | -0.66 |
| Thailand* | 0.04±0.21 | 0.00±0.00 | 0.00 | 0.21 | 0.21 |
| Ukraine* | 0.18±0.38 | 0.14±0.35 | 0.12 | 0.09 | 0.16 |
| Rest of Europe* | 0.45±0.50 | 0.17±0.38 | 0.24 | 0.56 | 0.42 |
| **Naive** |  |  |  |  |  |
| Male sex | 0.45±0.50 | 0.45±0.50 | 0.46 | 0.01 | -0.03 |
| Black ethnicity | 0.67±0.47 | 0.63±0.48 | 0.71 | 0.07 | -0.09 |
| Age at regimen start (years) | 13.34±3.32 | 11.12±3.16 | 13.03 | 0.67 | 0.09 |
| Prior AIDS diagnosis | 0.08±0.27 | 0.06±0.23 | 0.08 | 0.07 | -0.02 |
| UK/Ireland* | 0.31±0.46 | 0.61±0.49 | 0.67 | -0.65 | -0.78 |
| Thailand* | 0.09±0.29 | 0.00±0.00 | 0.00 | 0.31 | 0.31 |
| Ukraine* | 0.05±0.22 | 0.25±0.44 | 0.18 | -0.90 | -0.57 |
| Rest of Europe* | 0.55±0.50 | 0.14±0.34 | 0.15 | 0.83 | 0.80 |
| **ART experienced, VL≥200c/mL** |  |  |  |  |  |
| Male sex | 0.37±0.48 | 0.50±0.50 | 0.30 | -0.29 | 0.13 |
| Black ethnicity | 0.46±0.50 | 0.71±0.46 | 0.41 | -0.49 | 0.10 |
| Age at regimen start (years) | 13.82±3.04 | 13.13±3.04 | 13.95 | 0.23 | -0.04 |
| Prior AIDS diagnosis | 0.26±0.44 | 0.28±0.45 | 0.27 | -0.06 | -0.04 |
| Previous treatment failure | 0.46±0.50 | 0.52±0.50 | 0.47 | -0.11 | -0.00 |
| Time since ART start | 8.09±5.14 | 7.99±4.66 | 8.15 | 0.02 | -0.01 |
| UK/Ireland* | 0.28±0.45 | 0.74±0.44 | 0.59 | -1.01 | -0.68 |
| Thailand* | 0.06±0.24 | 0.00±0.00 | 0.00 | 0.25 | 0.25 |
| Ukraine* | 0.21±0.41 | 0.09±0.29 | 0.14 | 0.29 | 0.16 |
| Rest of Europe* | 0.45±0.50 | 0.17±0.38 | 0.27 | 0.55 | 0.36 |
| **ART experienced, VL<200c/mL** |  |  |  |  |  |
| Male sex | 0.52±0.50 | 0.48±0.50 | 0.49 | 0.08 | 0.04 |
| Black ethnicity | 0.57±0.50 | 0.74±0.44 | 0.59 | -0.34 | -0.04 |
| Age at regimen start (years) | 12.91±3.03 | 12.50±3.08 | 12.83 | 0.14 | 0.03 |
| Prior AIDS diagnosis | 0.24±0.43 | 0.24±0.43 | 0.22 | 0.01 | 0.05 |
| Previous treatment failure | 0.18±0.39 | 0.38±0.49 | 0.19 | -0.51 | -0.03 |
| Time since ART start | 8.69±4.20 | 8.19±3.88 | 8.55 | 0.12 | 0.03 |
| UK/Ireland* | 0.40±0.49 | 0.76±0.43 | 0.71 | -0.74 | -0.64 |
| Thailand* | 0.02±0.13 | 0.00±0.00 | 0.00 | 0.14 | 0.14 |
| Ukraine* | 0.11±0.31 | 0.03±0.16 | 0.06 | 0.25 | 0.15 |
| Rest of Europe* | 0.48±0.50 | 0.21±0.41 | 0.23 | 0.53 | 0.50 |
| **ART experienced, VL unknown** |  |  |  |  |  |
| Male sex | 0.47±0.50 | 0.47±0.50 | 0.36 | 0.01 | 0.24 |
| Black ethnicity | 0.29±0.45 | 0.52±0.50 | 0.36 | -0.51 | -0.15 |
| Age at regimen start (years) | 13.21±2.76 | 12.00±3.01 | 12.83 | 0.44 | 0.14 |
| Prior AIDS diagnosis | 0.19±0.39 | 0.24±0.43 | 0.22 | -0.13 | -0.08 |
| Previous treatment failure | 0.15±0.36 | 0.34±0.48 | 0.18 | -0.54 | -0.09 |
| Time since ART start | 9.43±3.66 | 5.38±3.92 | 9.21 | 1.11 | 0.06 |
| UK/Ireland* | 0.23±0.42 | 0.57±0.50 | 0.50 | -0.81 | -0.65 |
| Thailand* | 0.07±0.26 | 0.00±0.00 | 0.00 | 0.28 | 0.28 |
| Ukraine* | 0.37±0.48 | 0.33±0.47 | 0.21 | 0.10 | 0.33 |
| Rest of Europe* | 0.33±0.47 | 0.11±0.31 | 0.29 | 0.47 | 0.08 |
| *For continuous variables, standardised differences in means were provided while for binary variables absolute difference in percentage was shown. Standardised mean difference was calculated as the difference between the mean for those on DTG and the unweighted, then weighted, mean for those on comparator regimens, divided by the standard deviation for DTG. Propensity scores (PS) were estimated separately for those who were naive, ART experienced with VL≥200c/mL, ART experienced with VL<200c/mL and ART experienced unknown VL using logistic regression adjusting for key characteristics hypothesised as most likely to be associated with outcomes and shown in the table. Patients on comparator regimens were then weighted, with weights calculated as PS/(1-PS), to balance these characteristics. *All variables shown were included in the PS models apart from Region. Inclusion of region did not provide adequate balance across regions and led to imbalance in other characteristics. | | | | | |


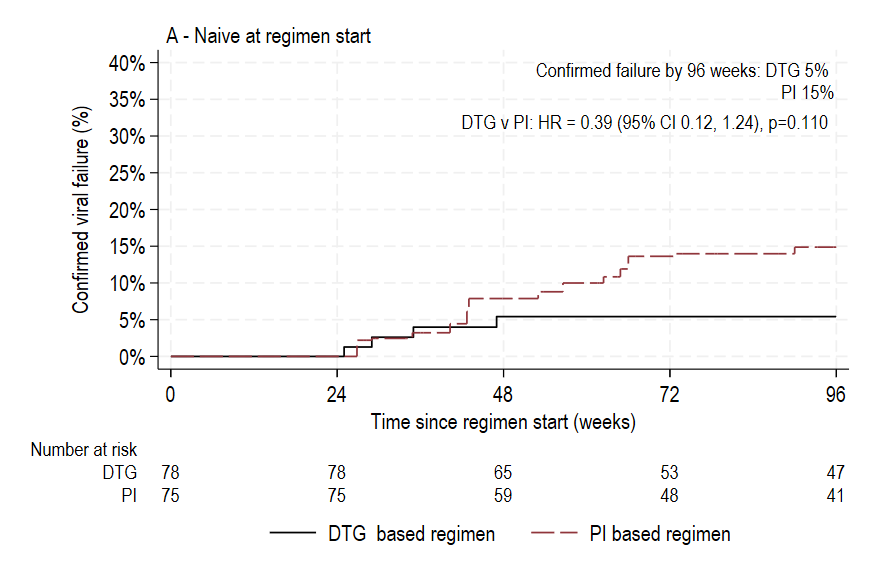

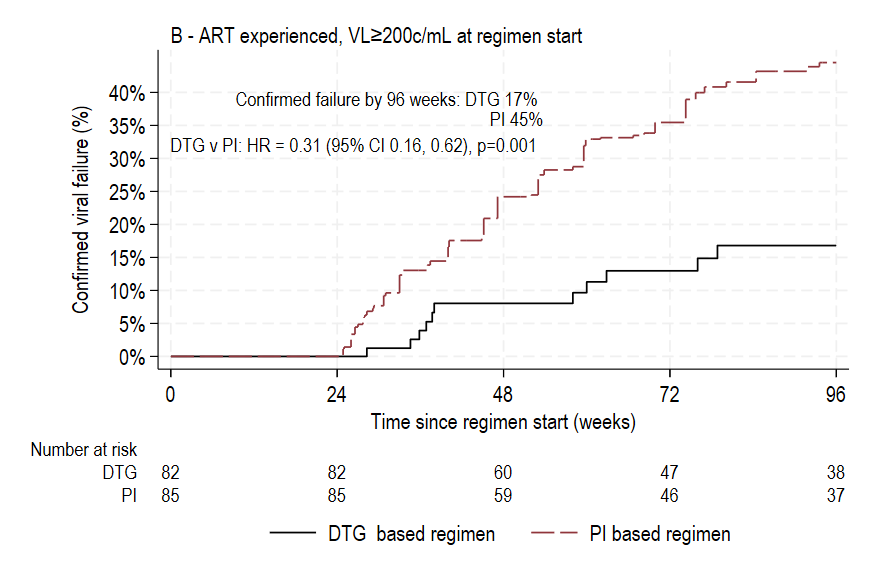

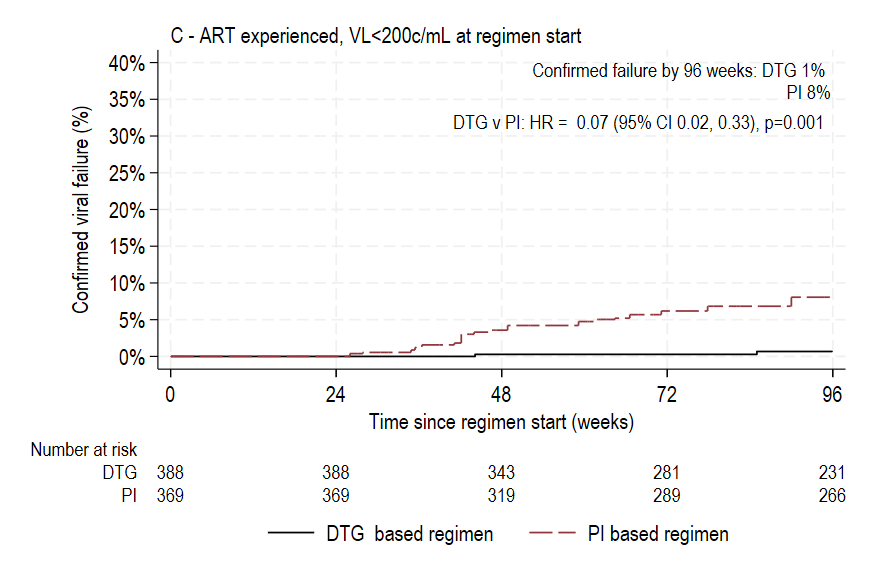

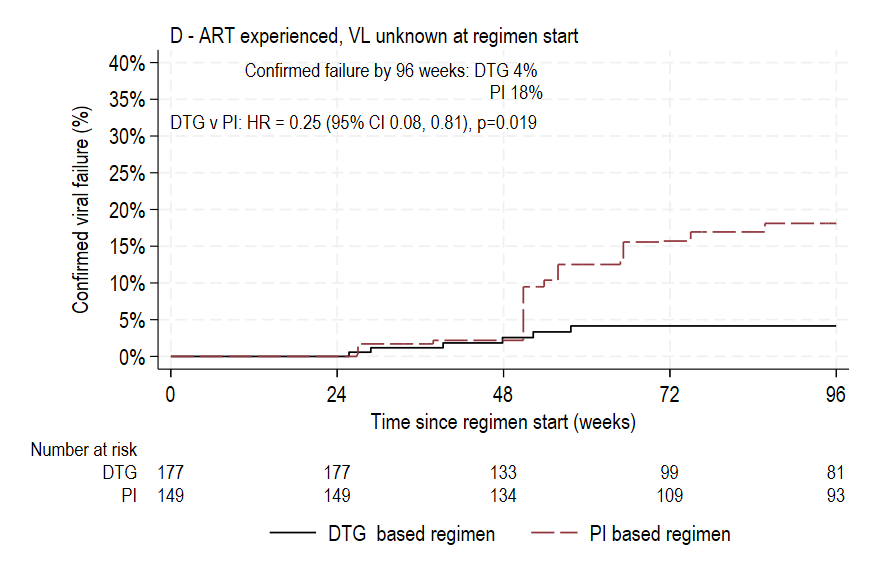


Viral failure was defined as 2 consecutive viral loads ≥400 copies/mL after 24 weeks of treatment OR 1 VL ≥400 copies/mL after 24 weeks of treatment followed by discontinuation within 4 months. For patients on comparator regimens the cumulative incidence function was weighted using propensity scores (PS) to balance key characteristics between comparator regimens and DTG (age, ethnicity (black versus other/unknown), sex and prior AIDS diagnosis (Yes versus no/unknown) (all patients) and time on ART and previous treatment failure (ART experienced only)). PSs were estimated separately for those who were naive, ART experienced with VL≥200c/mL, ART experienced with VL<200c/mL and ART experienced unknown VL with at least 24 weeks follow up after start of the regimen using logistic regression. Patients on PI regimens were weighted to reflect characteristics of those on DTG and effective number at risk after weighting is shown in the risk table. Hazard ratios and p-values for differences between DTG and comparator regimens were estimated using weighted Cox models.

**Figure S3: Time to viral failure on DTG- and PI-based regimens by ART and viral load status at DTG/PI start: (a) ART naïve, (b) ART experienced, VL ≥200c/mL, (c) ART experienced, VL <200c/mL and (d) ART experienced, VL unknown**

**Table S7. SAEs which occurred while on a DTG-based regimen (n=10)**

| **Number** | **Description** | **Discontinued DTG, reason (time after event onset, days)** |
| --- | --- | --- |
| **SAEs considered possibly, probably or definitively related to DTG (n=5)** | | |
| 1 | Headache during hospitalisation for a CNS lymphoma* | Yes, simplified treatment available (10 days) |
| 2 | Grade-3 raised creatinine during hospitalisation for rhabdomyolysis* | Yes, patient’s wish/decision (49 days) |
| 3 | Acute psychosis | Yes, unknown reason (6 days) |
| 4 | Grade 4 renal colic+renal stones+urolithiasis | No, still on drug at last follow-up visit |
| 5 | Grade 3 headache and tiredness | Yes, toxicity (0 days) |
| **SAEs with unknown relationship to DTG (n=3)** | | |
| 6 | Possible immune constitution inflammatory syndrome (IRIS); hospitalisation; diagnosis of viral meningitis | No, still on drug at last follow-up visit |
| 7 | Hospitalisation diagnosis – Bell's palsy, 7^th^ nerve palsy | Yes, unknown reason (265 days) |
| 8 | Grade 3 alkaline phosphatase; hospitalisation diagnosis – atypical mycobacterial infection not mycobacterium intracellulare | No, still on drug at last follow-up visit |
| **SAEs not causally related to DTG (n=2)** | | |
| 9 | Dermatoid cysts with inflammation and resorption; surgical operation | Yes, depressed mood (1158 days) |
| 10 | Hodgkin lymphoma | No, still on drug at last follow-up visit |

* Two of the SAEs related to DTG occurred during hospitalisation for another cause and were considered related to DTG by the treating physician. We could not confirm if the event resulted in prolonging hospitalization. Given this uncertainty, we opted for a cautious approach and classified them as SAEs.

**Table S8. Frequency and rates of first DAIDS grade laboratory events on DTG.**

|  | **No. of DTG episodes (patients)** | **Total no. events (tests)** | **Total no. grade ≥3 events (tests)*** | **DAIDS grade lab events on DTG (rate of first event per 100 patient years)** | | | | | | | | |  |
| --- | --- | --- | --- | --- | --- | --- | --- | --- | --- | --- | --- | --- | --- |
|  |  |  |  | **Grade 1** | | | **Grade 2** | | | **Grade ≥3†** | | |  |
|  |  |  |  | **Events** | **PY in 100s** | **Rate (95% CI)** | **Events** | **PY in 100s** | **Rate (95% CI)** | **Events** | **PY in 100s** | **Rate (95% CI)** |  |
| CHOL | 759 (750) | 292 (3049) | 3 (3) | 147 | 13 | 11.1 (9.4, 13.1) | 75 | 17 | 4.4 (3.5, 5.6) | 3 | 20 | 0.1 (0.0, 0.4) |  |
| HDL§ | 469 (460) | 226 (2203) | 0 (0) | 74 | 11 | 6.9 (5.4, 8.7) | 97 | 12 | 8.0 (6.5, 9.7) | - | - | N/A§ |  |
| LDL | 446 (439) | 146 (2079) | 5 (10) | 66 | 12 | 5.7 (4.4, 7.2) | 34 | 13 | 2.5 (1.7, 3.5) | 5 | 15 | 0.3 (0.1, 0.8) |  |
| TRIG | 713 (705) | 180 (2873) | 6 (7) | 119 | 16 | 7.3 (6.0, 8.7) | 19 | 19 | 1.0 (0.6, 1.6) | 6 | 20 | 0.3 (0.1, 0.7) |  |
| FPG | 148 (147) | 20 (526) | 0 (0) | 14 | 4 | 3.2 (1.7, 5.3) | 4 | 5 | 0.8 (0.2, 2.1) | 0 | 5 | 0.0 (0.0, 0.7) |  |
| NONFPG | 82 (81) | 7 (276) | 0 (0) | 6 | 2 | 2.7 (1.0, 5.9) | 1 | 2 | 0.4 (0.0, 2.3) | 0 | 2 | 0.0 (0.0, 1.5) |  |
| ANC | 755 (746) | 96 (3966) | 8 (8) | 42 | 17 | 2.4 (1.7, 3.3) | 14 | 18 | 0.8 (0.4, 1.3) | 8 | 19 | 0.4 (0.2, 0.8) |  |
| Hb | 805 (794) | 74 (4656) | 11 (66) | 37 | 19 | 1.9 (1.3, 2.6) | 13 | 20 | 0.6 (0.3, 1.1) | 10 | 20 | 0.5 (0.2, 0.9) |  |
| PLT | 721 (709) | 20 (4581) | 1 (1) | 10 | 20 | 0.5 (0.2, 0.9) | 3 | 20 | 0.2 (0.0, 0.4) | 1 | 20 | 0.1 (0.0, 0.3) |  |
| ALT | 515 (506) | 60 (3366) | 4 (4) | 43 | 13 | 3.3 (2.4, 4.4) | 8 | 14 | 0.6 (0.2, 1.1) | 4 | 14 | 0.3 (0.1, 0.7) |  |
| AMY | 63 (63) | 5 (352) | 0 (0) | 5 | 2 | 2.4 (0.8, 5.5) | 0 | 2 | 0.0 (0.0, 1.6) | 0 | 2 | 0.0 (0.0, 1.6) |  |
| APT | 388 (379) | 32 (2422) | 0 (0) | 23 | 12 | 1.9 (1.2, 2.9) | 5 | 13 | 0.4 (0.1, 0.9) | 0 | 13 | 0.0 (0.0, 0.3) |  |
| AST | 406 (399) | 66 (2580) | 2 (2) | 42 | 11 | 3.7 (2.7, 5.0) | 7 | 12 | 0.6 (0.2, 1.2) | 2 | 12 | 0.2 (0.0, 0.6) |  |
| GGT | 343 (337) | 40 (2169) | 1 (10) | 24 | 10 | 2.5 (1.6, 3.7) | 3 | 11 | 0.3 (0.1, 0.8) | 1 | 11 | 0.1 (0.0, 0.5) |  |
| LIP | 80 (79) | 2 (403) | 0 (0) | 1 | 3 | 0.3 (0.0, 1.6) | 1 | 3 | 0.3 (0.0, 1.6) | 0 | 3 | 0.0 (0.0, 1.1) |  |
| PHOS | 311 (307) | 92 (1355) | 2 (4) | 60 | 9 | 6.9 (5.2, 8.8) | 11 | 10 | 1.1 (0.5, 1.9) | 2 | 11 | 0.2 (0.0, 0.7) |  |
| SCa | 304 (300) | 60 (1472) | 5 (8) | 18 | 10 | 1.9 (1.1, 2.9) | 1 | 10 | 0.1 (0.0, 0.5) | 4 | 10 | 0.4 (0.1, 1.0) |  |
| SCREAT | 493 (484) | 147 (3204) | 2 (188) | 69 | 12 | 6.0 (4.6, 7.5) | 33 | 13 | 2.5 (1.8, 3.6) | 2 | 14 | 0.1 (0.0, 0.5) |  |
| TBIL | 449 (440) | 40 (2864) | 6 (13) | 17 | 14 | 1.2 (0.7, 1.9) | 9 | 14 | 0.6 (0.3, 1.2) | 5 | 15 | 0.3 (0.1, 0.8) |  |
| Note: Event rates were calculated for episodes on a DTG-based regimen (CALHIV could have more than one episode if they discontinued DTG for >30 days and then restarted DTG).  * Total number of grade ≥3 events may be higher than the number of grade 3 or 4 events for the rate calculations as the rate calculations only count first events. † For grade 3 and 4 events follow-up time was censored at the first grade 3 event, and if there was no grade 3 event then at the first grade 4 event. § There are no DAIDS grades for HDL, instead the guidelines from US Agency for Healthcare Research and Quality were used. 'Borderline low' is presented as grade 1, and 'low' as grade 2. There is no grade 3 or 4 category for HDL. Abbreviations: ALT, alanine aminotransferase; ANC, absolute neutrophil count; APT, alkaline phosphatase; AST, aspartate aminotransferase; CHOL, total cholesterol; CI, confidence interval; DTG, dolutegravir; FPG, fasting blood glucose; GGT, gamma glutamyl transferase; Hb, haemoglobin; HDL, high density lipoprotein cholesterol; LDL, low density lipoprotein cholesterol; LIP, lipase; m, month; NONFPG, non-fasting blood glucose; AMY, pancreatic amylase; PHOS, serum phosphate; PLT, platelets; PY, person years; SCa, serum calcium; SCREAT, serum creatinine; TBIL, total bilirubin; TRIG, triglycerides. | | | | | | | | | | | | | |

**
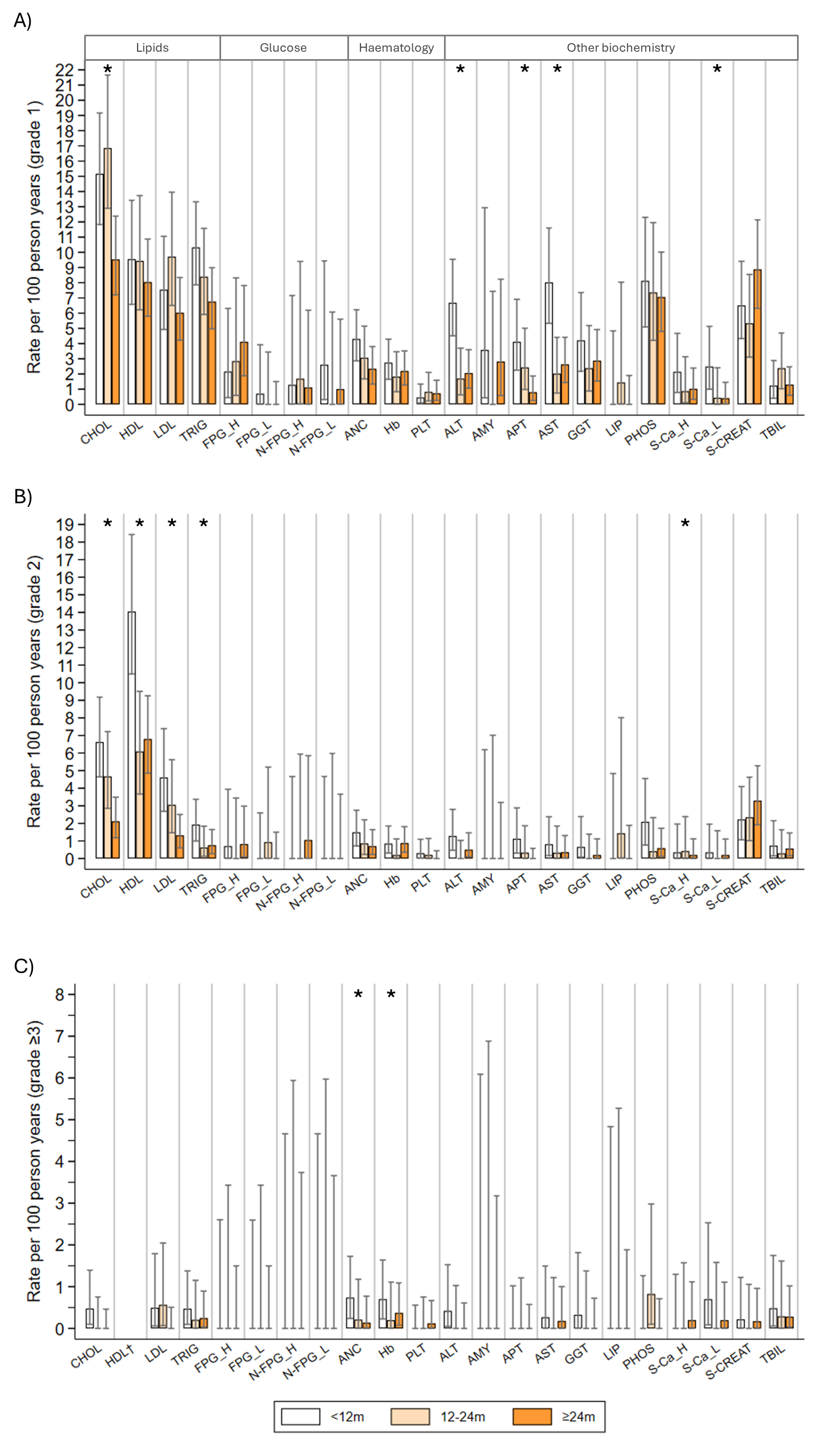
**

**Figure S4.** **Rates of first DAIDS grade laboratory events by time from DTG start and grade: A) grade 1, B) grade 2, and C) grade ≥3 events.**

Note: The white to dark orange bars reflect time periods after DTG start (<12; 12-24; ≥24 months). Events were calculated per episode on DTG. CALHIV could have more than one episode on DTG.

* P-value assessing difference across time categories was <0.05 (note: difference across time categories was only assessed if there was ≥1 event in each time category for that marker). † No definition for grade ≥3 HDL event.

**Abbreviations**: ALT, alanine aminotransferase; ANC, absolute neutrophil count; APT, alkaline phosphatase; AST, aspartate aminotransferase; CHOL, total cholesterol; CI, confidence interval; DTG, dolutegravir; FPG_L, low fasting blood glucose; FPG_H, high fasting blood glucose; GGT, gamma glutamyl transferase; Hb, haemoglobin; HDL, high density lipoprotein cholesterol; LDL, low density lipoprotein cholesterol; LIP, lipase; m, month; N-FPG_L, low non-fasting blood glucose; N-FPG_H, high non-fasting blood glucose; PAMY, pancreatic amylase; PHOS, serum phosphate; PLT, platelets; PY, person years; S-Ca_L, low serum calcium; S-Ca_H, high serum calcium; S-CREAT, serum creatinine; TBIL, total bilirubin; TRIG, triglycerides.

**Table S9. Rates of first laboratory events by grade for children and adolescents on DTG stratified by laboratory marker and time from DTG start.**

|  | No. of DTG episodes (patients) | Total no. events (tests) | Total no. grade ≥3 events (tests)* | DAIDS grade lab events on DTG† (rate of first event per 100 patient years) | | | | | | | | |
| --- | --- | --- | --- | --- | --- | --- | --- | --- | --- | --- | --- | --- |
|  |  |  |  | Grade 1 | | | Grade 2 | | | Grade ≥3‡ | | |
|  |  |  |  | Events | PY in 100s | Rate (95% CI) | Events | PY in 100s | Rate (95% CI) | Events | PY in 100s | Rate (95% CI) |
| CHOL |  |  |  |  |  |  |  |  |  |  |  |  |
| <12m | 651 (642) | 118 (1229) | 3 (3) | 70 | 5 | 15.2 (11.8, 19.2) | 36 | 5 | 6.6 (4.6, 9.2) | 3 | 6 | 0.5 (0.1, 1.4) |
| 12-24m | 434 (432) | 88 (759) | 0 (0) | 61 | 4 | 16.9 (12.9, 21.7) | 20 | 4 | 4.7 (2.9, 7.2) | 0 | 5 | 0.0 (0.0, 0.8) |
| >24m | 324 (323) | 86 (1055) | 0 (0) | 56 | 6 | 9.5 (7.2, 12.4) | 15 | 7 | 2.1 (1.2, 3.5) | 0 | 8 | 0.0 (0.0, 0.5) |
|  |  |  |  |  |  |  |  |  |  |  |  |  |
| HDL§ |  |  |  |  |  |  |  |  |  |  |  |  |
| <12m | 382 (373) | 87 (770) | 0 (0) | 33 | 3 | 9.6 (6.6, 13.4) | 52 | 4 | 14.0 (10.5, 18.4) | - | - | N/A§ |
| 12-24m | 306 (304) | 47 (544) | 0 (0) | 27 | 3 | 9.4 (6.2, 13.7) | 19 | 3 | 6.1 (3.7, 9.5) | - | - | N/A§ |
| >24m | 259 (258) | 91 (885) | 0 (0) | 42 | 5 | 8.0 (5.8, 10.9) | 40 | 6 | 6.8 (4.9, 9.3) | - | - | N/A§ |
|  |  |  |  |  |  |  |  |  |  |  |  |  |
| LDL |  |  |  |  |  |  |  |  |  |  |  |  |
| <12m | 363 (356) | 49 (747) | 3 (8) | 26 | 3 | 7.5 (4.9, 11.0) | 17 | 4 | 4.6 (2.7, 7.4) | 2 | 4 | 0.5 (0.1, 1.8) |
| 12-24m | 290 (289) | 42 (511) | 2 (2) | 29 | 3 | 9.7 (6.5, 14.0) | 10 | 3 | 3.1 (1.5, 5.6) | 2 | 4 | 0.6 (0.1, 2.0) |
| >24m | 248 (247) | 55 (817) | 0 (0) | 36 | 6 | 6.0 (4.2, 8.3) | 9 | 7 | 1.3 (0.6, 2.5) | 0 | 7 | 0.0 (0.0, 0.5) |
|  |  |  |  |  |  |  |  |  |  |  |  |  |
| TRIG |  |  |  |  |  |  |  |  |  |  |  |  |
| <12m | 601 (593) | 77 (1148) | 3 (3) | 59 | 6 | 10.3 (7.9, 13.3) | 12 | 6 | 1.9 (1.0, 3.4) | 3 | 6 | 0.5 (0.1, 1.4) |
| 12-24m | 411 (409) | 42 (716) | 1 (1) | 37 | 4 | 8.4 (5.9, 11.6) | 3 | 5 | 0.6 (0.1, 1.8) | 1 | 5 | 0.2 (0.0, 1.2) |
| >24m | 315 (314) | 61 (1003) | 2 (3) | 47 | 7 | 6.8 (5.0, 9.0) | 6 | 8 | 0.8 (0.3, 1.7) | 2 | 8 | 0.2 (0.0, 0.9) |
|  |  |  |  |  |  |  |  |  |  |  |  |  |
| FPG |  |  |  |  |  |  |  |  |  |  |  |  |
| <12m | 112 (111) | 5 (175) | 0 (0) | 4 | 1 | 2.9 (0.8, 7.4) | 1 | 1 | 0.7 (0.0, 4.0) | 0 | 1 | 0.0 (0.0, 2.6) |
| 12-24m | 69 (69) | 4 (108) | 0 (0) | 3 | 1 | 2.9 (0.6, 8.3) | 1 | 1 | 0.9 (0.0, 5.2) | 0 | 1 | 0.0 (0.0, 3.4) |
| >24m | 71 (71) | 11 (243) | 0 (0) | 9 | 2 | 4.1 (1.9, 7.8) | 2 | 2 | 0.8 (0.1, 3.0) | 0 | 2 | 0.0 (0.0, 1.5) |
|  |  |  |  |  |  |  |  |  |  |  |  |  |
| NONFPG |  |  |  |  |  |  |  |  |  |  |  |  |
| <12m | 49 (48) | 3 (112) | 0 (0) | 3 | 1 | 4.0 (0.8, 11.6) | 0 | 1 | 0.0 (0.0, 4.7) | 0 | 1 | 0.0 (0.0, 4.7) |
| 12-24m | 35 (34) | 1 (69) | 0 (0) | 1 | 1 | 1.7 (0.0, 9.6) | 0 | 1 | 0.0 (0.0, 6.0) | 0 | 1 | 0.0 (0.0, 6.0) |
| >24m | 36 (36) | 3 (93) | 0 (0) | 2 | 1 | 2.3 (0.3, 8.1) | 1 | 1 | 1.0 (0.0, 5.8) | 0 | 1 | 0.0 (0.0, 3.7) |
|  |  |  |  |  |  |  |  |  |  |  |  |  |
| ANC |  |  |  |  |  |  |  |  |  |  |  |  |
| <12m | 689 (680) | 44 (1609) | 5 (5) | 28 | 7 | 4.3 (2.9, 6.2) | 10 | 7 | 1.5 (0.7, 2.7) | 5 | 7 | 0.7 (0.2, 1.7) |
| 12-24m | 436 (433) | 20 (952) | 1 (1) | 14 | 5 | 3.1 (1.7, 5.1) | 4 | 5 | 0.9 (0.2, 2.2) | 1 | 5 | 0.2 (0.0, 1.2) |
| >24m | 306 (305) | 31 (1399) | 1 (1) | 16 | 7 | 2.3 (1.3, 3.8) | 5 | 7 | 0.7 (0.2, 1.6) | 1 | 7 | 0.1 (0.0, 0.8) |
|  |  |  |  |  |  |  |  |  |  |  |  |  |
| Hb |  |  |  |  |  |  |  |  |  |  |  |  |
| <12m | 752 (741) | 33 (1868) | 7 (17) | 19 | 7 | 2.7 (1.7, 4.3) | 6 | 7 | 0.9 (0.3, 1.9) | 5 | 7 | 0.7 (0.2, 1.6) |
| 12-24m | 499 (496) | 11 (1115) | 1 (33) | 9 | 5 | 1.8 (0.8, 3.5) | 1 | 5 | 0.2 (0.0, 1.1) | 1 | 5 | 0.2 (0.0, 1.1) |
| >24m | 340 (339) | 30 (1667) | 3 (16) | 17 | 8 | 2.2 (1.3, 3.5) | 7 | 8 | 0.9 (0.4, 1.8) | 3 | 8 | 0.4 (0.1, 1.1) |
|  |  |  |  |  |  |  |  |  |  |  |  |  |
| PLT |  |  |  |  |  |  |  |  |  |  |  |  |
| <12m | 641 (629) | 6 (1749) | 0 (0) | 3 | 7 | 0.5 (0.1, 1.3) | 2 | 7 | 0.3 (0.0, 1.1) | 0 | 7 | 0.0 (0.0, 0.6) |
| 12-24m | 458 (455) | 5 (1094) | 0 (0) | 4 | 5 | 0.8 (0.2, 2.1) | 1 | 5 | 0.2 (0.0, 1.1) | 0 | 5 | 0.0 (0.0, 0.8) |
| >24m | 350 (349) | 9 (1734) | 1 (1) | 6 | 8 | 0.7 (0.3, 1.6) | 0 | 8 | 0.0 (0.0, 0.4) | 1 | 8 | 0.1 (0.0, 0.7) |
|  |  |  |  |  |  |  |  |  |  |  |  |  |
| ALT |  |  |  |  |  |  |  |  |  |  |  |  |
| <12m | 478 (469) | 39 (1288) | 3 (3) | 30 | 4 | 6.7 (4.5, 9.5) | 6 | 5 | 1.3 (0.5, 2.8) | 2 | 5 | 0.4 (0.1, 1.5) |
| 12-24m | 342 (341) | 6 (826) | 0 (0) | 6 | 4 | 1.7 (0.6, 3.7) | 0 | 4 | 0.0 (0.0, 1.0) | 0 | 4 | 0.0 (0.0, 1.0) |
| >24m | 254 (253) | 15 (1248) | 0 (0) | 12 | 6 | 2.1 (1.1, 3.6) | 3 | 6 | 0.5 (0.1, 1.5) | 0 | 6 | 0.0 (0.0, 0.6) |
|  |  |  |  |  |  |  |  |  |  |  |  |  |
| AMY |  |  |  |  |  |  |  |  |  |  |  |  |
| <12m | 52 (52) | 2 (109) | 0 (0) | 2 | 1 | 3.6 (0.4, 12.9) | 0 | 1 | 0.0 (0.0, 6.2) | 0 | 1 | 0.0 (0.0, 6.1) |
| 12-24m | 43 (43) | 0 (87) | 0 (0) | 0 | 0 | 0.0 (0.0, 7.4) | 0 | 1 | 0.0 (0.0, 7.0) | 0 | 1 | 0.0 (0.0, 6.9) |
| >24m | 37 (37) | 3 (156) | 0 (0) | 3 | 1 | 2.8 (0.6, 8.2) | 0 | 1 | 0.0 (0.0, 3.2) | 0 | 1 | 0.0 (0.0, 3.2) |
|  |  |  |  |  |  |  |  |  |  |  |  |  |
| APT |  |  |  |  |  |  |  |  |  |  |  |  |
| <12m | 352 (344) | 19 (924) | 0 (0) | 14 | 3 | 4.1 (2.3, 6.9) | 4 | 4 | 1.1 (0.3, 2.9) | 0 | 4 | 0.0 (0.0, 1.0) |
| 12-24m | 252 (251) | 8 (582) | 0 (0) | 7 | 3 | 2.4 (1.0, 5.0) | 1 | 3 | 0.3 (0.0, 1.9) | 0 | 3 | 0.0 (0.0, 1.2) |
| >24m | 188 (187) | 5 (912) | 0 (0) | 5 | 6 | 0.8 (0.3, 1.9) | 0 | 6 | 0.0 (0.0, 0.6) | 0 | 6 | 0.0 (0.0, 0.6) |
|  |  |  |  |  |  |  |  |  |  |  |  |  |
| AST |  |  |  |  |  |  |  |  |  |  |  |  |
| <12m | 371 (364) | 36 (954) | 1 (1) | 28 | 3 | 8.0 (5.3, 11.6) | 3 | 4 | 0.8 (0.2, 2.4) | 1 | 4 | 0.3 (0.0, 1.5) |
| 12-24m | 285 (284) | 8 (623) | 0 (0) | 6 | 3 | 2.0 (0.7, 4.4) | 1 | 3 | 0.3 (0.0, 1.8) | 0 | 3 | 0.0 (0.0, 1.2) |
| >24m | 217 (216) | 22 (1001) | 1 (1) | 14 | 5 | 2.6 (1.4, 4.4) | 2 | 6 | 0.4 (0.0, 1.3) | 1 | 6 | 0.2 (0.0, 1.0) |
|  |  |  |  |  |  |  |  |  |  |  |  |  |
| GGT |  |  |  |  |  |  |  |  |  |  |  |  |
| <12m | 307 (301) | 15 (726) | 1 (0) | 12 | 3 | 4.2 (2.2, 7.4) | 2 | 3 | 0.7 (0.1, 2.4) | 1 | 3 | 0.3 (0.0, 1.8) |
| 12-24m | 247 (246) | 6 (502) | 0 (0) | 6 | 3 | 2.4 (0.9, 5.2) | 0 | 3 | 0.0 (0.0, 1.4) | 0 | 3 | 0.0 (0.0, 1.4) |
| >24m | 197 (196) | 19 (937) | 0 (10) | 13 | 5 | 2.9 (1.5, 4.9) | 1 | 5 | 0.2 (0.0, 1.1) | 0 | 5 | 0.0 (0.0, 0.7) |
|  |  |  |  |  |  |  |  |  |  |  |  |  |
| LIP |  |  |  |  |  |  |  |  |  |  |  |  |
| <12m | 65 (64) | 0 (131) | 0 (0) | 0 | 1 | 0.0 (0.0, 4.8) | 0 | 1 | 0.0 (0.0, 4.8) | 0 | 1 | 0.0 (0.0, 4.8) |
| 12-24m | 52 (52) | 2 (99) | 0 (0) | 1 | 1 | 1.4 (0.0, 8.0) | 1 | 1 | 1.4 (0.0, 8.0) | 0 | 1 | 0.0 (0.0, 5.3) |
| >24m | 47 (47) | 0 (173) | 0 (0) | 0 | 2 | 0.0 (0.0, 1.9) | 0 | 2 | 0.0 (0.0, 1.9) | 0 | 2 | 0.0 (0.0, 1.9) |
|  |  |  |  |  |  |  |  |  |  |  |  |  |
| PHOS |  |  |  |  |  |  |  |  |  |  |  |  |
| <12m | 230 (226) | 32 (553) | 0 (0) | 22 | 3 | 8.1 (5.1, 12.3) | 6 | 3 | 2.1 (0.8, 4.5) | 0 | 3 | 0.0 (0.0, 1.3) |
| 12-24m | 164 (164) | 21 (342) | 2 (4) | 16 | 2 | 7.4 (4.2, 12.0) | 1 | 2 | 0.4 (0.0, 2.3) | 2 | 2 | 0.8 (0.1, 3.0) |
| >24m | 158 (158) | 39 (460) | 0 (0) | 31 | 4 | 7.1 (4.8, 10.0) | 3 | 5 | 0.6 (0.1, 1.7) | 0 | 5 | 0.0 (0.0, 0.7) |
|  |  |  |  |  |  |  |  |  |  |  |  |  |
| SCa |  |  |  |  |  |  |  |  |  |  |  |  |
| <12m | 229 (226) | 21 (588) | 2 (2) | 12 | 3 | 4.4 (2.3, 7.6) | 1 | 3 | 0.4 (0.0, 2.0) | 2 | 3 | 0.7 (0.1, 2.5) |
| 12-24m | 169 (169) | 16 (364) | 0 (3) | 2 | 2 | 0.9 (0.1, 3.2) | 1 | 2 | 0.4 (0.0, 2.4) | 0 | 2 | 0.0 (0.0, 1.6) |
| >24m | 156 (156) | 23 (520) | 3 (3) | 6 | 5 | 1.2 (0.5, 2.7) | 1 | 5 | 0.2 (0.0, 1.1) | 2 | 5 | 0.4 (0.0, 1.5) |
|  |  |  |  |  |  |  |  |  |  |  |  |  |
| SCREAT |  |  |  |  |  |  |  |  |  |  |  |  |
| <12m | 451 (442) | 42 (1205) | 1 (40) | 28 | 4 | 6.5 (4.3, 9.4) | 10 | 4 | 2.2 (1.1, 4.1) | 1 | 5 | 0.2 (0.0, 1.2) |
| 12-24m | 335 (334) | 27 (800) | 0 (65) | 17 | 3 | 5.3 (3.1, 8.5) | 8 | 3 | 2.3 (1.0, 4.6) | 0 | 3 | 0.0 (0.0, 1.1) |
| >24m | 245 (244) | 77 (1195) | 1 (83) | 39 | 4 | 8.9 (6.3, 12.1) | 17 | 5 | 3.3 (1.9, 5.3) | 1 | 6 | 0.2 (0.0, 1.0) |
|  |  |  |  |  |  |  |  |  |  |  |  |  |
| TBIL |  |  |  |  |  |  |  |  |  |  |  |  |
| <12m | 408 (400) | 12 (1091) | 3 (8) | 5 | 4 | 1.2 (0.4, 2.9) | 3 | 4 | 0.7 (0.2, 2.1) | 2 | 4 | 0.5 (0.1, 1.7) |
| 12-24m | 311 (310) | 11 (679) | 1 (3) | 8 | 3 | 2.4 (1.0, 4.7) | 1 | 3 | 0.3 (0.0, 1.6) | 1 | 3 | 0.3 (0.0, 1.6) |
| >24m | 245 (244) | 17 (1090) | 2 (2) | 9 | 7 | 1.3 (0.6, 2.5) | 4 | 7 | 0.6 (0.2, 1.5) | 2 | 7 | 0.3 (0.0, 1.0) |
| Note: Event rates were calculated for episodes on a DTG-based regimen (CALHIV could have more than one episode if they discontinued DTG for >30 days and then restarted DTG).  * Total number of grade ≥3 events may be higher than the number of grade 3 or 4 events for the rate calculations as the rate calculations only count first events. † Number of episodes with ≥1 test results in each grade. It is not possible to add the number of events in each time period to equal the overall number in Table S8 as the same child can contribute follow-up time to >1 time period. Each child/episode is only counted once in each grade (with grades 3 and 4 counted together) in each time period, to prevent an artificial overestimation of rates due to increased testing during an event. For the calculation of rates, follow-up time is censored at the beginning of the first event of that grade or higher within each time period. ‡ For grade 3 and 4 events follow-up time is censored at the first grade 3 event, and if there is no grade 3 event then at the first grade 4 event. § There are no DAIDS grades for HDL, instead the guidelines from US Agency for Healthcare Research and Quality were used. 'Borderline low' is presented as grade 1, and 'low' as grade 2. There is no grade 3 or 4 category for HDL. Abbreviations: ALT, alanine aminotransferase; ANC, absolute neutrophil count; APT, alkaline phosphatase; AST, aspartate aminotransferase; CHOL, total cholesterol; CI, confidence interval; DTG, dolutegravir; FPG, fasting blood glucose; GGT, gamma glutamyl transferase; Hb, haemoglobin; HDL, high density lipoprotein cholesterol; LDL, low density lipoprotein cholesterol; LIP, lipase; m, month; NONFPG, non-fasting blood glucose; AMY, pancreatic amylase; PHOS, serum phosphate; PLT, platelets; PY, person years; SCa, serum calcium; SCREAT, serum creatinine; TBIL, total bilirubin; TRIG, triglycerides. | | | | | | | | | | | | |


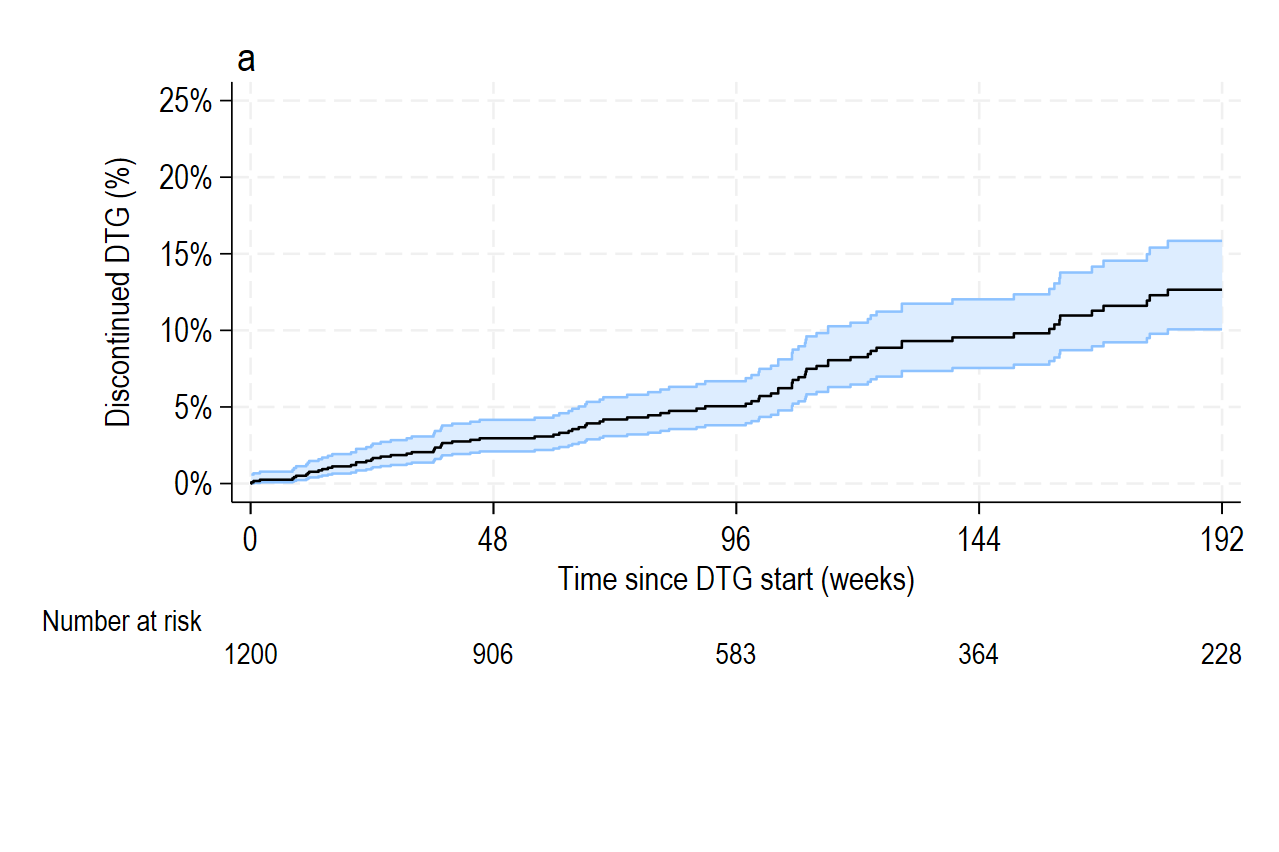

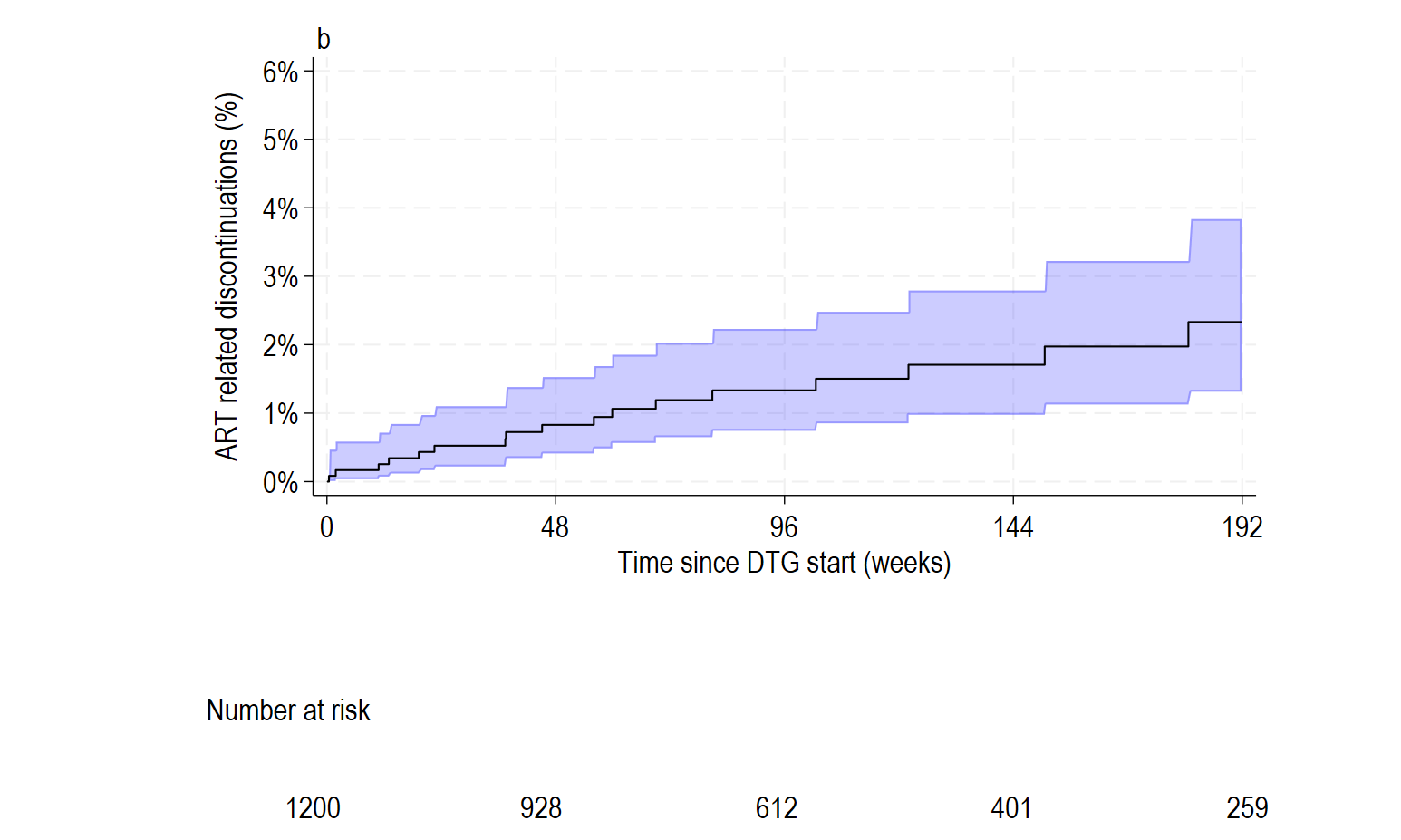


Discontinuation was defined as stopping DTG ≥30 days. Cumulative incidence of discontinuation was estimated using Kaplan Meier methods. Follow up was censored at last visit. Patients who started DTG in a trial were excluded.

**Figure S5: Time to DTG discontinuation overall (a) all cause discontinuation and (b) treatment-related discontinuation (with discontinuations for other reasons treated as a competing risk)**

| **Table S10: Reasons for discontinuation of DTG** | | |
| --- | --- | --- |
|  | **N** | **%** |
| **Treatment related discontinuations (n=22)** | | |
| Virological failure | 5 | 5 |
| Hypersensitivity reaction | 1 | 1 |
| Toxicity - gastrointestinal tract | 3 | 3 |
| Toxicity - haematological | 1 | 1 |
| Toxicity - kidney | 2 | 2 |
| Toxicity - nervous system | 1 | 1 |
| Toxicity - neuropsychiatric | 2 | 2 |
| Toxicity - pancreas | 1 | 1 |
| Toxicity - other | 5 | 5 |
| Unwanted weight changes | 1 | 1 |
| **Other reasons (n=73)** | | |
| Simplified/more effective treatment available | 17 | 18 |
| Structured Treatment Interruption | 1 | 1 |
| Physician's decision | 7 | 7 |
| Patient's wish | 11 | 12 |
| Non-compliance | 4 | 4 |
| Drug not available | 2 | 2 |
| Other | 3 | 3 |
| Unknown | 28 | 29 |
| Total | 95 | 100 |
